# Effects on Colonization Factors and Mechanisms Involved in Antimicrobial Sonophotodynamic Inactivation Mediated by Curcumin

**DOI:** 10.3390/pharmaceutics15102407

**Published:** 2023-09-30

**Authors:** Fernanda Alves, Sebastião Pratavieira, Natália Mayumi Inada, Claudia Patricia Barrera Patiño, Cristina Kurachi

**Affiliations:** Instituto de Física de São Carlos, Universidade de São Paulo, São Carlos, São Paulo CEP 13560-970, Brazil; prata@ifsc.usp.br (S.P.); nataliainada@ifsc.usp.br (N.M.I.); cpbarrerap@ifsc.usp.br (C.P.B.P.); cristina@ifsc.usp.br (C.K.)

**Keywords:** sonophotodynamic therapy, biofilm, virulence factors, *Staphylococcus aureus*

## Abstract

Photodynamic (PDI) and sonodynamic (SDI) inactivation have been successfully employed as antimicrobial treatments. Moreover, sonophotodynamic inactivation (SPDI), which is the simultaneous application of PDI and SDI, has demonstrated greater effects. This study assessed the effects of PDI (PDI group), SDI (SDI group) and SPDI (SPDI group) using curcumin as a sensitizer on the metabolism, adhesion capability, biofilm formation ability and structural effects in a *Staphylococcus aureus* biofilm. Moreover, the production of reactive oxygen species (ROS) and the degradation spectrum of curcumin under the irradiation sources were measured. SPDI was more effective in inactivating the biofilm than PDI and SDI. All treatments reduced the adhesion ability of the bacteria: 58 ± 2%, 58 ± 1% and 71 ± 1% of the bacterial cells adhered to the polystyrene plate after the SPDI, SDI and PDI, respectively, when compared to 79 ± 1% of the untreated cells (control group). This result is probably related to the metabolism cell reduction after treatments. The metabolism of cells from the PDI group was 89 ± 1% lower than the untreated cells, while the metabolic activity of SDI and SPDI groups were 82 ± 2% and 90 ± 1% lower, respectively. Regarding the biofilm formation ability, all treatments (SPDI, SDI and PDI) reduced the total biomass. The total biomass of the PDI, SDI and SPDI groups were 26 ± 2%, 31 ± 5% and 35 ± 6% lower than the untreated biofilm (control group), respectively. Additionally, all treatments produced ROS and caused significant structural changes, reducing cells and the extracellular matrix. The light caused a greater absorbance decay of the curcumin; however, the US did not expressively alter its spectrum. Finally, SPDI had improved antimicrobial effects, and all treatments exhibited similar effects in the colonization factors evaluated.

## 1. Introduction

*Staphylococcus aureus* is an important type of Gram-positive bacteria that lives in the human body in a commensal manner; however, under certain conditions (immunosuppression states, diabetes mellitus and elderly individuals), this microorganism causes superficial or deep infections, such as contaminated ulcers on the skin, pneumonia and sepsis. In the same way as other microorganisms, *S. aureus* has powerful tools to cause infectious diseases successfully, which are called virulence factors [1]. Virulence factors are cellular structures, molecules and regulatory systems that assist the bacterium to colonize the host tissue [2]. The biofilm living form of the microorganisms is considered an important virulence factor that helps them to establish a highly structured microbial community [3]. A biofilm is a complex microbial organization, where the microorganisms are embedded in a self-produced extracellular matrix (ECM) that protects them from chemical agents and physical stress [4]. The biofilm formation is an endless process, and this life cycle is divided into the following steps: (I) adhesion: microorganisms are reversibly adsorbed to a biotic or abiotic surface; (II) colonization: microorganisms are irreversibly attached to the surface; (III) development: the multilayered cells proliferate, and the ECM is produced and secreted; (IV) maturation: the formation of a three-dimensional stable community that contains channels to efficiently distribute nutrients and signaling molecules (quorum sensing) within the biofilm; and (V) dispersal: detachment of microbial cells to disseminate and, subsequently, colonize other sites [4]. The presence of the ECM that protects the cells, the quorum sensing molecules that are produced and the architecture of the biofilm make this community more resistant to antimicrobial therapies than the planktonic counterparts. Moreover, around 70% of infectious diseases are caused by microorganisms organized as biofilms, increasing to 85% when considering chronic infections [5]; for this reason, the management of biofilms is the main challenge for microbial control [4].

The need to develop new antimicrobial treatments to combat microbial biofilms is unquestionable. In this context, antimicrobial photodynamic inactivation (PDI) is a powerful treatment that has been studied and proposed worldwide, and its effectiveness against bacteria, fungi and viruses has been demonstrated in a series of in vitro, in vivo and clinical studies. The PDI is based on the combination of three compounds: a photosensitizer (PS), a light source at an appropriate wavelength to excite the PS and molecular oxygen in the target tissue [6]. The mechanisms involved in the PDI action are very well known and described. When the PS molecule in its singlet ground state is illuminated, it absorbs energy, enters a high-energy state and remains activated. During the PS relaxation process, highly cytotoxic reactive oxygen species (ROS) are generated by two reactions: type 1 and type 2. In a type 1 reaction, when the PS is in the triplet excited state can interact with oxygen or other substrate molecules directly and transfer a proton or an electron to the substrate to form a radical anion or radical cation, these radicals may react with oxygen to produce ROS, such as superoxide anion (O_2_^−^), hydrogen peroxide (H_2_O_2_) and hydroxyl radical (HO•). In a type 2 reaction, the PS can directly transfer energy to molecular oxygen producing the excited state singlet oxygen (^1^O_2_). Both type 1 and 2 reactions occur simultaneously, but depending on the PS chemical structure, one of the reactions will be preferential. The efficiency of the PDI is often related to the ^1^O_2_ quantum yield of the PS [6,7,8,9,10,11].

Interestingly, it has been verified that many of the available clinically used photosensitizers can be also activated through ultrasound (US) [12,13,14,15,16], even though the exact mechanism(s) by which this occurs remain(s) unknown. This antimicrobial strategy is called sonodynamic therapy (SDI). The SDI mechanism is governed by multiple factors depending on the nature of the biological model, the sonosensitizer and the ultrasound parameters [17]. The precise functioning of SDI mechanisms remains not completely comprehended, though they can be categorized into three primary phases [18]. To begin, ultrasound (US) must be generated in the region where sensitizer substances are present. Subsequently, sonochemical reactions are induced by the occurrence of cavitation and mechanical forces. Ultrasonic cavitation represents a singular and dynamic ultrasound phenomenon that acts upon the surrounding medium, resulting in the formation of microbubbles that undergo a sequence of excitation, vibration, contraction and, in certain instances, collapse. Depending on the intensity of the ultrasound, cavitation manifests in distinct ways, characterized as non-inertial cavitation (also referred to as stable cavitation) and inertial cavitation. Non-inertial cavitation arises when low-intensity ultrasound is applied within a liquid medium, leading to the production of bubbles that do not promptly collapse, exhibiting an extended lifespan. These non-inertial cavitation bubbles possess substantial energy, oscillate vigorously and are capable of generating radiation force and high-speed microjets. Consequently, they can interact with nearby entities like cells, biomolecules and structures, such as the cell membrane. When these microbubbles reach the cell membrane, they induce the formation of temporary pores, allowing the adjacent therapeutic agents, like sonosensitizers, to penetrate the cells.

In contrast, inertial cavitation takes place when ultrasound is applied to a liquid, resulting in a vigorous and dynamic bubble behavior. Inertial cavitation bubbles absorb substantial energy and release it in a confined area, causing an escalation in local temperature and pressure, the formation of free radicals, the generation of intense shock waves and the emergence of high-speed microjets within the medium. This physical scenario, combined with the generation of chemical radicals, proves highly detrimental to target cells, causing profound effects on their biomolecules. In essence, cavitation regimes trigger elevated temperatures and pressures, leading to the production of hydroxyl radicals and hydrogen atoms. Moreover, the high pressure and temperature can decompose solutes within the medium. Ultimately, the production of reactive oxygen species (ROS) during the collapse of bubbles initiates chemical reactions in the liquid. Another phenomenon associated with ROS production is sonoluminescence, which involves the emission of light resulting from the collapse of bubbles and is hypothesized to activate the sonosensitizer.

Additionally, a novel approach called sonophotodynamic therapy (SPDI) has been investigated to enhance microbial inactivation [19,20,21,22] by combining ultrasound (US) and light to activate the photosensitizer (PS). This strategy leverages the mechanical effects of US in conjunction with light’s ability to excite the PS. In the context of cancer treatment, SPDI is currently under evaluation, and studies have demonstrated that sensitizers can be effectively activated by both light and US sources, rendering the combined treatment (SPDI) more efficacious than isolated therapies (PDI or SDI) [19,23]. In the microbial field, this pattern of higher effectiveness of SPDI was also verified against *Candida albicans*, *Enterococcus faecalis*, *Aggregatibacter actinomycetemcomitans*, *Porphyromonas gingivalis Prevotella intermedia* [19,24,25,26].

As mentioned before, the sensitizers applied in PDI may be used to mediate SDI and SPDI [12,13,14,15,16]. One promising sensitizer to mediate PDI, SDI and SPDI is curcumin. Curcumin (molecular formula: C_21_H_20_O_6_) [27] is a natural compound, considered a consolidated photosensitizer demonstrating great results against Gram-positive and Gram-negative bacteria. Besides that, curcumin exhibits properties that confer safe and controlled administration in humans, i.e., it is inert, its bioaccumulation is within the standards, the reaction products with radiation are nontoxic, and its cytotoxicity for human cells is non-significant [28]. Recently, our research group proved that PDI, SDI and SPDI mediated by curcumin were effective in inactivating a *S. aureus* biofilm, and the highest reduction was achieved with the combined treatment (SPDI), where 7.43 log of inactivation was obtained [15]. Additionally, studies have evaluated the association of PDI (with curcumin) and other techniques to potentiate treatments [15]. In the attempt to better understand the effects and mechanisms involved in the bacteria inactivation, the present work evaluated the effects on the metabolism, adhesion and biofilm formation abilities of the bacteria *S. aureus* after the treatments, the production of reactive oxygen species with each therapy and the spectrum degradation of the curcumin over the sources of the treatments (light, US and light + US).

## 2. Materials and Methods

### 2.1. PS, Light Source and Ultrasound

The curcumin (PDI Pharma, Cravinhos, SP, Brazil) was used as photosensitizer. A stock solution of 16 mM was prepared in DMSO and then diluted in sterile saline to the final concentration of 40 μM (keeping the final concentration of DMSO at 0.25% *v*/*v*). For this study, a customized device combining a blue LED and ultrasound was developed at São Carlos Institute of Physics, University of São Paulo. The LED-based source has centered emission at 455 nm (LXHL-PR09, Luxeon^®^ III Emitter, Lumileds Lighting, San Jose, CA, USA), with an irradiance of 37 mW/cm^2^. The ultrasound, coupled in the same device, was used at a frequency of 1 MHz, pulse repetition frequency of 100 Hz, 20% of duty cycle and 3 W/cm^2^ of intensity.

### 2.2. Microorganism and Biofilm Formation

In this study, the Methicillin-sensitive *Staphylococcus aureus* (ATCC number 25923) bacterial strain was chosen for evaluation. To reactivate the bacteria that were stored at −20 °C in tubes containing Tryptic Soy Broth (TSB) mixed with 50% glycerol, they were cultured on brain heart infusion (BHI) agar plates and incubated at 37 °C for 24 h. Following this incubation period, 5–10 bacterial colonies were collected and suspended in a tube containing 10 mL of TSB. This suspension was then incubated at 37 °C for 16 h. Next, an aliquot of 500 μL from this suspension was diluted in 9.5 mL of fresh TSB. This diluted suspension was allowed to grow until the mid-log growth phase, and its optical density was standardized to 0.2 arbitrary units (a.u.) as determined by using a spectrophotometer (Varian Cary^®^ 50 UV-Vis Spectrophotometer—Agilent, Santa Clara, CA, USA), which corresponds to a concentration of 10^8^ cells/mL.

For the formation of bacterial biofilms, 1 mL of the standardized bacterial suspension was placed in cell culture Petri dishes (with a diameter of 34 mm) and incubated at 37 °C in a shaker incubator set at 75 rpm for 90 min (adhesion phase). Following this initial adhesion phase, the Petri dishes were washed twice with phosphate-buffered saline (PBS) to eliminate non-adhered cells. Subsequently, 1 mL of TSB was added to each Petri dish. The biofilms were allowed to develop by incubating them for 48 h in an orbital shaker (75 rpm). After this biofilm formation period, the suspension was removed, the biofilms were washed twice with PBS, and then we proceeded with the treatment.

### 2.3. Treatments

After 48 h of biofilm formation, samples were submitted to SDI, PDI or SPDI. In order to evaluate the effects of the treatments on the remaining bacteria, sub-lethal doses of the treatments were applied to recover live cells, and the parameters used were selected based on our previous work [15]. In this study [15], we observed that the parameters of curcumin at 80 μM, the application of US for 32 min with a power density of 3 W/cm^2^, 20% of a duty cycle and the blue LED light irradiation at the dose of 70 J/cm^2^ were able to significantly inactivate the *S. aureus* biofilm. For this reason, in the present work, the curcumin concentration, US and light parameters were lower than that, as described below. For the SDI (sonodynamic inactivation) group, 2 mL of curcumin (Cur) at a concentration of 40 μM was added, and the plates were kept in darkness for 20 min. Subsequently, an ultrasound (US) transducer was used on the biofilms, operating at a frequency of 1 MHz with a power density of 3 W/cm^2^, a duty cycle of 20% and a pulse frequency of 100 Hz for a duration of 15 min. For the PDI (photodynamic inactivation) group, biofilms were incubated for 20 min with 2 mL of Cur. Following this, blue LED light was directed at the bottom of the cell culture plates, delivering a dose of 35 J/cm^2^. In the SPDI (sonophotodynamic inactivation) group, biofilm samples were first incubated with curcumin for 20 min. Then, both light and ultrasound were applied simultaneously, using the same parameters as mentioned earlier. Other biofilm samples were subjected to individual treatments: sensitizer application only (Cur group), ultrasound exposure only (US group), LED light exposure only (Light group) or received no treatment (control group).

### 2.4. Evaluation Immediately after Treatments

To assess the impact of the treatments on the biofilms, the following methods were employed: quantification of colonies (CFU/mL), evaluation of cellular metabolism (XTT assay) and measurement of total biomass.

Colony quantification (CFU/mL) was carried out by detaching the biofilms by rubbing the pipette tip on the bottom of the Petri dish for 30 s. To assess cell survival, samples were serially diluted 10-fold in sterile saline, and duplicate 25 μL aliquots were spread on BHI agar plates. The plates were then incubated under aerobic conditions at 37 °C for 48 h, followed by the calculation of colony-forming units (CFU/mL). This process was performed in duplicate on three separate occasions (*n* = 6).

Biofilm cell metabolism was determined through the XTT assay, where 1 mL of XTT solution was added to each Petri dish after the treatments. Samples were incubated with the solution containing 790 μL of PBS with 200 mM glucose, 200 μL of XTT and 10 μL of menadione. Then, plates were incubated at 37 °C in darkness for 3 h and colorimetrically measured at 492 nm using a microplate reader (Thermo Plate/TP Reader).

The total biofilm biomass was quantified using crystal violet (CV) staining. After the treatments, biofilms were fixed with 1 mL of methanol for 15 min and then air-dried at 37 °C for 20 min. Subsequently, 1 mL of CV (1%, *v*/*v*) was added and allowed to sit for 5 min. After a wash with ultrapure water, 33% acetic acid was applied to remove the dye, and the resulting solubility was measured at 595 nm using a microplate reader.

### 2.5. Evaluation of the Adhesion Ability and Biofilm-Forming Capacity

Additional biofilms that had undergone the aforementioned treatments were analyzed. To assess adhesion ability, treated biofilms were detached from the Petri dishes, the cells were transferred to new Petri dishes, and the same procedures for adhesion of cells were followed. After adhesion, viability was determined using the CFU/mL assay, and these values were statistically compared with those obtained from biofilms immediately after treatments.

To evaluate the biofilm-forming capacity, biofilms subjected to the treatments were detached from the Petri dishes, and microorganisms underwent the same procedures as described previously for biofilm development. After biofilm formation, colony quantification (CFU/mL) and total biomass measurements were performed. The resulting values were then statistically compared with those obtained from biofilms immediately after treatments.

### 2.6. Confocal Fluorescence Microscopy

The effects of the treatments on the biofilm components (bacterial cells and extracellular matrix) were examined using confocal fluorescence microscopy (LSM780, Carl Zeiss, Oberkochen, Germany). After the treatments, the Cur solution was removed, and the biofilm was washed twice with saline. The fluorescent LIVE/DEAD Baclight Bacterial Viability Kit was utilized as per the manufacturer’s instructions. This kit uses SYTO 9 and Propidium Iodide (PI) dyes, with SYTO 9 staining viable bacterial cells (green fluorescence) and PI marking nonviable bacterial cells (red fluorescence). After staining, the biofilms were washed twice with sterile saline. The extracellular matrix (ECM) was stained with the blue fluorescent dye Calcofluor White (Sigma Aldrich^®^, St. Louis, MO, USA), which binds to glycans and can detect extracellular polysaccharides. Biofilms were incubated with 50 μg/mL of Calcofluor for 10 min. The stained biofilms were then imaged using recommended excitation/emission wavelengths: 480/500 nm for SYTO-9 stain, 490/635 nm for PI and 405/433 nm for Calcofluor.

### 2.7. Optical Coherence Tomography (OCT)

The OCT imaging was performed to visualize the effect of each treatment on the biofilm structure. For this, after treatments, the Cur was removed, biofilms were carefully washed twice with saline solution, and then samples were observed in the OCT equipment (ThorLabs, Model Tel300, Telesto series, Probe.uni, nominal center length of 1310 nm). Images were obtained with medium sensitivity, a speed of 76 kHz, field image correction, a subsampling filter, a scanning pattern of 400 × 400 × 512 pixels (X, Y, Z) and a pixel size of 5 × 5 × 2.49 μm (X, Y, Z). Transversal images of the biofilms were obtained to evaluate the cell density, thickness and topography of the biofilm.

### 2.8. Reactive Oxygen Species Generation

To evaluate the production of oxygen singlet species and hydroxyl radicals by SDI, PDI and SPDI mediated by curcumin, the fluorescent probes APF (Hydroxyl Radical, Hypochlorite or Peroxynitrite Sensor, which exhibits bright green fluorescence, with excitation/emission maxima ∼490/515 nm) and SOSG (Singlet Oxygen Sensor Green Reagent, which in the presence of singlet oxygen emits green fluorescence, with excitation/emission maxima ~504/525 nm), both from Thermo Fisher (Waltham, MA, USA), were used. The stock solution of each probe was diluted in phosphate buffer according to the manufacturer’s instructions at the final concentration of 3 μM. The treatments were applied in the presence of the probes individually. Control groups consisted of the US, light, US + light and Cur only. The fluorescence was measured using the spectrophotometer (Cary Eclipse Fluorescence spectrophotometer, Agilient (Santa Clara, CA, USA) at the appropriate wavelength for each probe. These experiments were performed on 3 different occasions, with 3 samples on each occasion.

### 2.9. Curcumin Degradation over Treatments

To evaluate the Cur degradation over each treatment, the absorbance spectrum of Cur was monitored in a spectrophotometer (Varian Cary^®^ 50 UV-Vis Spectrophotometer—Agilent, Santa Clara, CA, USA). For this, the curcumin was prepared at the concentration of 32 μM in DMSO, and it was exposed to the sources of each treatment: light (PDI), US (SDI) and light + US (SPDI), and the absorbance spectrum was obtained every 5 min of exposition until 30 min. These experiments were performed on 3 different occasions, with 3 samples on each occasion.

### 2.10. Statistical Analyses

The CFU/mL values were converted into logarithmic (log10) form, and we assessed the uniformity of variance and the normal distribution of the data using the Levene and Shapiro–Wilk tests, respectively. Statistical analysis was conducted through a one-way analysis of variance (one-way ANOVA), and to compare multiple groups, the post hoc Tukey test was employed (with a significance level of α = 0.05). These statistical procedures were carried out using the SPSS software package (IBM^®^ SPSS^®^ Statistics, version 20, Chicago, IL, USA).

## 3. Results and Discussion

**Figure 1:**
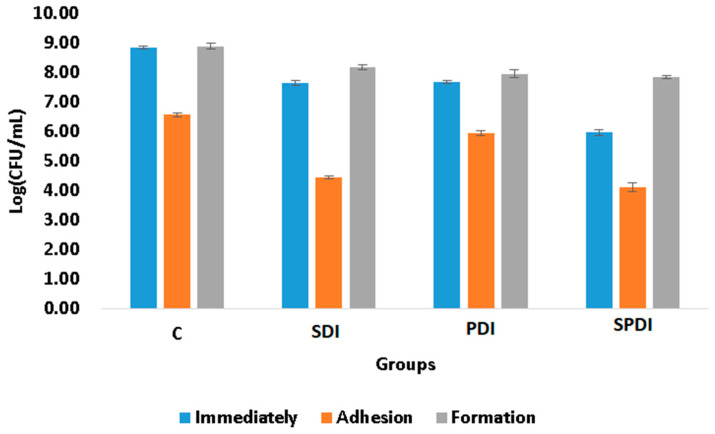
Mean log values (CFU/mL) and standard deviation of the control (C), SDI, PDI and SPDI groups at the different periods of evaluation (immediately after the treatments, after adhesion and post biofilm formation).

The adhesion ability of the microorganisms to biotic or abiotic surfaces, such as mucosa, ulcers, prostheses, dental implants and catheters, is of great importance, since it is the first step of the free cells to form the biofilm community and, consequently, establish a successful microbial community and infection. The complexity of the bacterial tools used for cell adhesion and invasion ranges from single monomeric proteins to intricate multimeric macromolecules that perform highly sophisticated functions. The surface organelles and toxins secreted allow the pathogenic bacteria to invade many different niches throughout the course of an infection. In the present study, the effect of PDI, SDI and SPDI on the ability of the bacteria to attach (adhere) to an abiotic surface was evaluated. Firstly, the treatment parameters were selected in order to obtain an effect on bacteria and also to be able to collect the survived cells for the posterior analyses of adhesion and biofilm formation ability. The SDI, PDI and SPDI groups exhibited 1 ± 1., 1 ± 1 and 3 ± 1 log of reduction, respectively, in comparison with the control group. After determining the parameters of the treatments, the effects on the adhesion and biofilm formation ability were assessed. According to the viability assay (CFU/mL), it was observed that the sub-lethal dose of the treatments was able to decrease the adhesion ability of *S. aureus* (Figure 1). Analyzing the treatments individually, from the survived cells of the SPDI group, 58 ± 2% of the bacteria were able to adhere to the polystyrene plate, so 42 ± 2% of the surviving cells were not able to attach to the surface (Table 1). For the SDI group, 58 ± 1% of the bacteria also had capacity for adherence. In the PDI group, 71 ± 1 of the bacteria attached to the plate surface. In the control group (that did not receive any treatment), 21 ± 1% of the cells were not able to adhere to the polystyrene plate (Table 1). The Cur, US and only Light groups were similar to the control group (*p* = 0.999) and were not included in Figure 1. To the best of our knowledge, this is the first study that evaluated the action of SDI and SPDI on adhesion ability, as other studies investigated only the PDI effects. The work of Xin Li et al. [29] evaluated the effects of toluidine blue O (TBO)-mediated photodynamic therapy on *Staphylococcus epidermidis* adherence and biofilm formation using confocal laser scanning microscopy. The results of the adhesion assay indicated that the control groups exhibited significant bacterial adherence compared with the TBO-PDI groups. Analysis of the biofilm formation revealed significant light dose-dependent differences between the TBO-PDI groups and the control groups [29]. Alves et al. [30] evaluated the interference of PDI mediated by Photodithazine on the adhesion ability of *Candida albicans* biofilm in vitro. The authors did not verify a significant difference in the capacity for adhesion of the cells treated with PDI in comparison with the other groups [30]. In the study conducted by Soares et al. [31], where they investigated the impact of PDI using TBO on the ability of *Candida* species to adhere to bucco-epithelial cells (BECs), their findings indicated a direct relationship: as the efficacy of PDI against *Candida* species increased, there was a corresponding decrease in the yeast’s adherence to BECs [31].

The biofilm formation ability of some bacteria species is also considered a pathogenicity factor and resistance mechanism that protects the microorganisms from physical, chemical and environmental stress. A biofilm represents a structured community of microorganisms residing within a self-generated extracellular matrix (ECM). This matrix primarily consists of polysaccharides, proteins, lipids and nucleic acids (such as RNA and extracellular DNA). Together, these components create a highly moist, polar blend that forms the fundamental framework and three-dimensional structure of a biofilm. The biofilm is regarded as one of the most successful forms of microbial existence on Earth and is the prevailing microbial lifestyle in natural settings. Additionally, research has revealed that a significant portion—around 70–75%—of human infections are associated with microorganisms organized in the form of biofilms, with this living form being more resistant to the treatments than the planktonic counterpart [4]. In the present study, the biofilm formation ability was evaluated after the treatments. For this, the cells that survived the treatments were re-submitted to the biofilm formation steps and, after 48 h of maturation, the viability assay was performed to quantify the cells in the biofilm. It was observed that none of the therapies were able to alter the biofilm formation ability, since the same number of living cells collected immediately after the treatments was also obtained after 48 h of biofilm formation (Figure 1), following the same pattern of the control group. The Cur, US and only Light groups were also similar to the control group (*p* = 0.999) and were not included in Figure 1. In the literature, the biofilm ability has been only evaluated after PDI, and the results obtained depend on the target microorganism and the PS/light used. Carmello et al. [32] showed that the PDI mediated by chloro-aluminum phthalocyanine encapsulated in cationic nanoemulsion was capable of reducing the biofilm ability of *Candida albicans* present on oral candidosis of mice [32]. In another study, *Candida krusei* also exhibited a reduction in this virulence factor after being treated by PDI mediated by toluidine blue [33]. Moreover, PDI mediated by methylene blue reduced the biofilm formation ability of *Serratia marcescens* [34]. Finally, sub-lethal doses of PDI mediated by TBO, methylene blue and indocyanine green affected the biofilm formation ability and metabolic activity of *Enterococcus faecalis* [35]. However, Alves et al. [30] demonstrated that PDI mediated by Photodithazine did not alter the adherence and biofilm formation ability of fluconazole-susceptible and fluconazole-resistant *C. albicans* [30].

**Figure 2:**
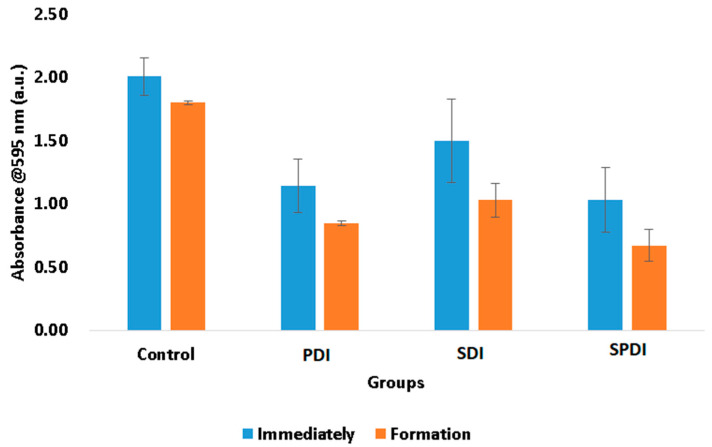
Mean values and standard deviation of the total biomass of the control, SDI, PDI and SPDI groups at different periods of evaluation (immediately after the treatments and post biofilm formation).

The total biomass of the biofilms at the end of the therapies and after 48 h of biofilm formation were also evaluated by means of a crystal violet assay. This measurement represents both bacteria cells and the ECM involving them. In this test, it was observed that the treatments were able to reduce the biomass immediately after in comparison with the control group. The PDI samples exhibited a reduction in the total biomass equivalent to 43 ± 9%, the SDI group exhibited a reduction of 25 ± 11%, and the SPDI group exhibited a reduction of 49 ± 11% in comparison with the control group (Figure 2). After the treatments, the survived cells were re-submitted to the biofilm formation steps, and the total biomass was once again evaluated. It was observed that all groups (including the control group) demonstrated a reduction in the total biomass in comparison with the values obtained at the “immediately” period of evaluation (Figure 2). The control group showed a total biomass 10 ± 1% lower than that found in the immediate period. The PDI group demonstrated a reduction of 26 ± 2%, the SDI showed a reduction of 31 ± 5%, and the SPDI group showed a reduction of 35 ± 6% of the total biomass in comparison with the immediate period of evaluation of each group (Figure 2, Table 1). With these results, it is possible to conclude that PDI, SDI and SPDI treatments were capable of reducing the biomass and that the biofilm formed by the survivor cells was thinner. This means that the treatments significantly changed the biofilm characteristics, mainly in the SPDI group. Taking the CFU/mL assay into account, it is possible to correlate the total biomass results with those found in the viability test. The CFU/mL assay showed no change in the values in the biofilm formed after the treatments; however, the total biomass was reduced, and for this reason, it is possible to conclude that the reduction in the crystal violet measurement was mainly in the extracellular matrix of the biofilm. This is an important result, since a thinner biofilm with a lesser amount of ECM involving the cells makes the bacteria more susceptible to the next antimicrobial therapy.

**Figure 3:**
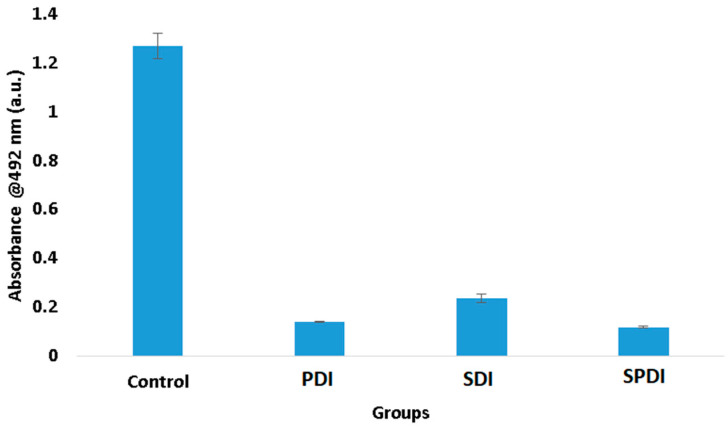
Mean values and standard deviation of the metabolic activity of the control, SDI, PDI and SPDI groups immediately after the treatments.

In trying to understand these results of viability and total biomass of the biofilm, we performed the XTT assay, which evaluates the metabolic activity of the cells. According to the results obtained, it was observed that the three therapies expressively reduced the cell metabolism. The PDI group exhibited a metabolic activity 89 ± 1% lower than the control group, while SDI and SPDI showed metabolic activity 82 ± 2% and 90 ± 1% lower, respectively (Figure 3, Table 1). This reduction totally influences biofilm development. *Staphylococcal* biofilm development is a complex process that is divided into phases, such as the initial attachment, production of the extracellular matrix, cell proliferation, biofilm structuring and cell detachment. In all of these steps, there are many biological events, such as gene expression, enzyme production and secretion, cell multiplication (growth and division) and cell detachment. All of these cell events are dependent on the cell metabolism status. For this reason, cells with a low metabolism have deficient biofilm development. The results of the present study demonstrated that the therapies reduced the metabolism, and it is possible to conclude that the biofilm development was hindered, explaining the reduction in the total biomass.

**Figure 4:**
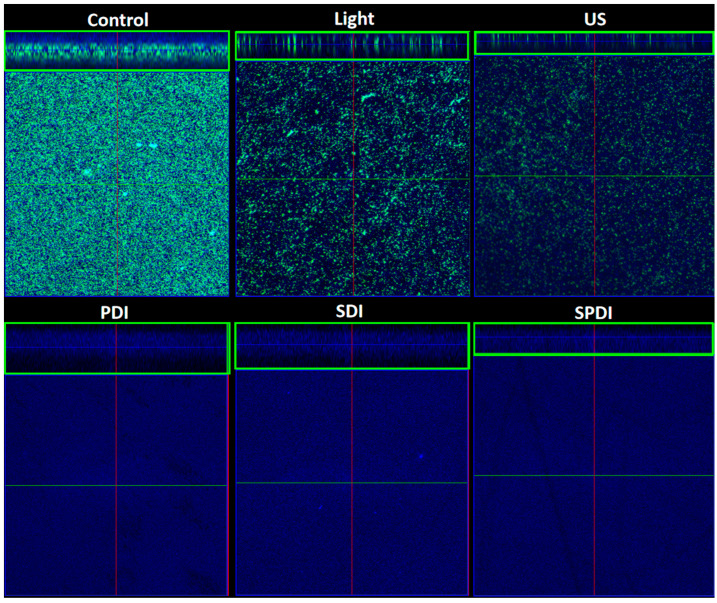
Confocal images obtained of the biofilms of the control, light, US, PDI, SDI and SPDI groups. Biofilms were stained with LIVE/DEAD (SYTO 9 and Propidium Iodide), which marks viable (green color) and nonviable cells (red color). The ECM was stained with Calcofluor White (blue color). The stained biofilms were imaged using excitation/emission wavelengths at 480/500 nm for the SYTO-9 stain, 490/635 nm for PI and 405/433 nm for Calcofluor, as recommended by the manufacturers. The green boxes are transversal images showing the thickness of the biofilms.

**Figure 5:**
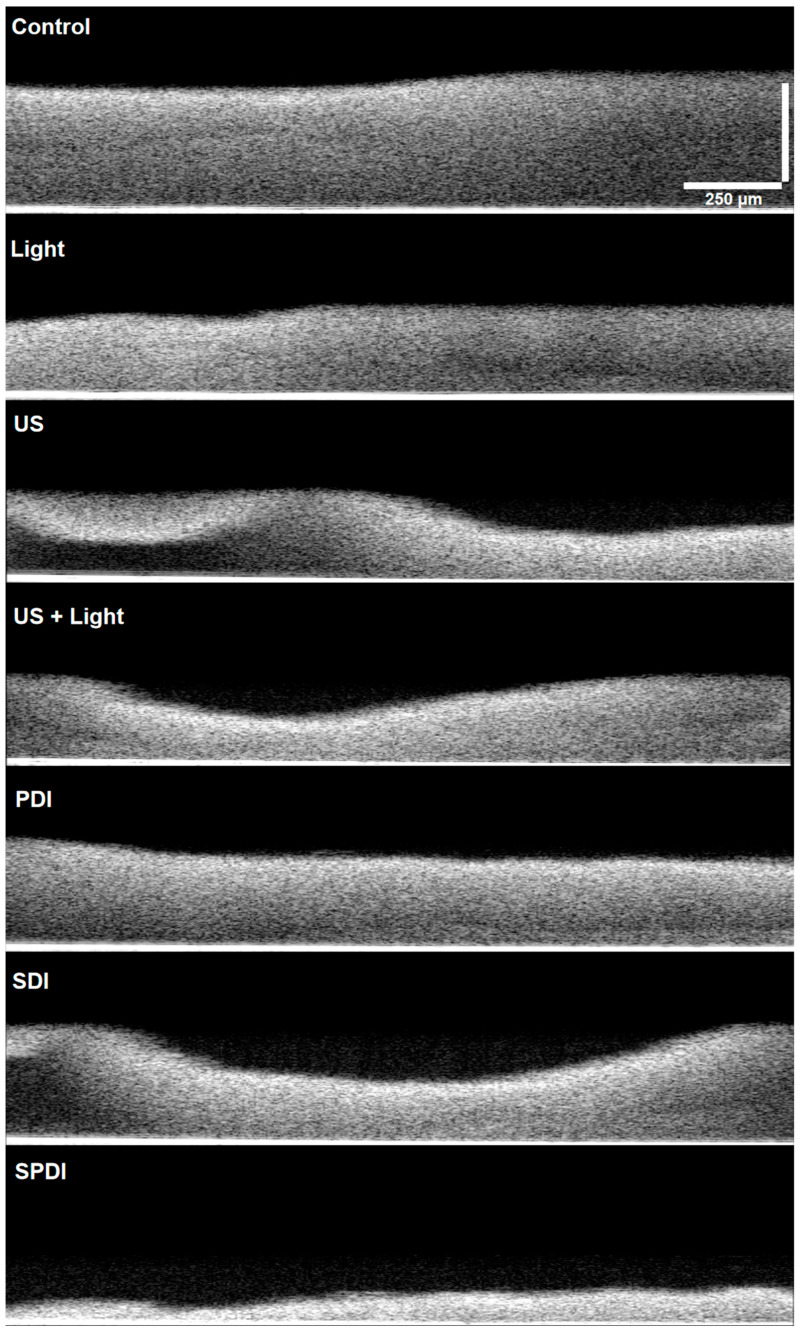
OCT images obtained from the Control, light US, US + Light, PDI, SDI and SPDI groups. Images were obtained with medium sensitivity, speed of 76 kHz, field image correction, subsampling filter, scanning pattern of 400 × 400 × 512 pixels (X, Y, Z) and pixel size of 5 × 5 × 2.49 μm (X, Y, Z). Transversal images of the biofilms were obtained to evaluate the cell density, thickness and topography of the biofilm. The scale bar of 250 μm present on the top of the image, it is the same for all groups.

The effects of the treatments on the biofilm components were evaluated under confocal microscopy, where live/dead cells and the ECM were stained after treatments (Figure 4, and raw images are in Figure S1). According to the images obtained, all treatments caused a high impact on the bacteria cells, where no live or dead cells were detected. It is important to emphasize that no live nor dead cells were observed in the PDI, SDI and SPDI groups, since they were detached from the biofilm because of the action of the treatments, and after removing the fluorochromes from the wells for the microscopic analysis, cells were not detected [36]. Additionally, there was a reduction in the amount of ECM of all treated groups (PDI, SDI and SPDI) when compared with the control group. Regarding the effects of light or ultrasound alone (without Cur), a lesser effect of these sources on the cells and ECM amount was observed; however, a less populated biofilm with some defects was observed. Additionally, transversal images were also obtained in confocal microscopy (green boxes in Figure 4), and it is possible to observe that no cell can be detected from the top to the bottom of the biofilm submitted to all treatments. Finally, the control group was full of live cells embedded in the ECM, covering the entire analyzed area. These results all together show the ability of the dynamic process involved in the treatments (PDI, SDI and SPDI) to cause a significant impact on the biofilm components, reducing the cells and ECM. Additionally, the biofilm structure was assessed by using OCT (Figure 5 and raw images are in Figure S2). This assay revealed that PDI and Light groups showed very similar characteristics to each other, such as density and topography, and exhibited a lesser reduction in the thickness compared to the control. The US, US + light and SDI groups exhibited a more expressive alteration, with lower thickness compared to the control, defects on the topography and some regions with lower biomass density. However, the most impacted biofilm was the SPDI group, where a destructive effect on the structure of the biofilm was observed and a thin layer of biomass was imaged.

These results evidence the mechanical action of the ultrasound on the biofilm by itself, where the US, US + Light and SDI groups exhibited structural alterations, and this effect was extremely enhanced when the three components (sensitizer, ultrasound and light) were applied together (SDPT group). The mechanical effect of US is governed by the dynamic phenomenon called cavitation, where microbubbles with high energy interact with the media [17]. Depending on the parameters of the ultrasound (US), cavitation phenomena manifest differently, leading to a distinction between two types: non-inertial cavitation, also known as stable cavitation, and inertial cavitation. Non-inertial cavitation occurs when low-intensity ultrasound is applied in a liquid medium. This is characterized by the generation of high-energy bubbles that do not rapidly collapse. These bubbles can produce effects like radiation force, microstreaming, and a push–pull action. In contrast, inertial cavitation involves bubbles that absorb significant amounts of energy and release it within a confined space. This results in an increase in local temperature and pressure, the formation of free radicals, the emergence of powerful shockwaves, and the creation of high-speed micro-jets in the media [17]. All of these events interact with the structures that are close to them, such as the cells and the ECM, being able to cause transient or permanent structural alterations. An advantage of using the US to mediate the treatments is that its mechanical effects are nonspecific and could be applied to Gram-positive or Gram-negative bacteria, fungi and susceptible or resistant microorganisms, even causing severe alterations in biofilms, as demonstrated in the present study.

In the present work, the production of oxygen singlet species and hydroxyl radicals in response to each treatment was also evaluated by the use of fluorescent probes (SOSG and APF, respectively) in an attempt to correlate inactivation with ROS production. These results are shown in Table 2, where the mean fluoresce values and the standard deviation of all samples are present. The production of the reactive oxygen species by PDI, SDI and SPDI were dependent on the source of irradiation. In the groups where the US was applied, there was a predominance of hydroxyl radicals; on the other hand, when the light was used, the production of oxygen singlet species was observed (Table 2). These results showed that curcumin reacts differently to ultrasound and light. Presumably, when the ultrasound is applied, there is a preference for a type I reaction, so the PS in the T_1_ can transfer a proton or an electron to the substrate to form a radical anion or radical cation; these radicals may react with oxygen to produce ROS. On the opposite side, when the light is used, it facilitates a type 2 reaction, and then the PS in the T_1_ can directly transfer energy to molecular oxygen (a triplet in the ground state), producing the excited state singlet oxygen (^1^O_2_). It is important to emphasize that the SPDI group generated both singlet oxygen and hydroxyl radicals. The enhancement of ROS production by SPDI has been reported in the literature. Karanlık et al. [37] reported a sono–photochemical study of SPDI mediated by phthalocyanine-based sensitizers. The authors calculated the singlet oxygen quantum yield (ΦΔ) resulting from the singlet oxygen chemical quencher–1,3-diphenylisobenzofuran (DPBF) decomposition, and they obtained values equivalent to 0.68 for zinc-phthalocyanine and 0.83 for indium-phthalocyanine. Following exposure to light, these values rose to 0.81 and 0.94, respectively. Notably, when ultrasound (US) and light irradiation were combined, the values increased further. These findings clearly demonstrate a significant boost in singlet oxygen production when transitioning from light irradiation alone to the combined use of US and light. The increase in singlet oxygen yield can be attributed to two primary factors as suggested by the researchers. Firstly, there is a greater dispersion of oxygen (O_2_) and the photosensitizer in the medium. Secondly, the higher local pressures resulting from the combined approach lead to an increased frequency factor, which contributes to the observed enhancement in singlet oxygen production [37]. Additionally, the exact mechanism of the sonodynamic process for ROS production is unclear. However, it is suggested that ROS-dependent SDI mechanisms are initiated by inertial cavitation [38]. The generated sonoluminescence and the accompanying heat, ranging from 4000 to 25,000 Kelvin, trigger nearby sonosensitizers through a process similar to PDI or pyrolysis [39].

Another point that is important to highlight is the fact that when the US or light was used solely (in the absence of the Cur) but in the presence of the probes, there was a high production of hydroxyl radicals (by the US) and singlet oxygen (by the light), even more than in the treatment groups (PDI, SDI and SPDI). This may be explained by the fact that curcumin shows antioxidant action. Then, it is difficult to analyze ROS production properly when this sensitizer is involved, even when the production during the treatment is high. Moreover, absorption events may occur with curcumin during fluorescence measurement. Besides that, the ROS generated by the US and light were not high enough to induce bacteria inactivation and/or were not produced in close proximity to the bacteria cells. When considering biofilms, there is a high influence of extracellular polymeric substances [40,41]. The photosensitizer molecules may be trapped in the extracellular matrix, not being available to the bacterial cells, and the produced ROS may have other targets, not resulting in cell death. For this reason, dynamic therapies are more effective than irradiation with light or US only.

There is a limitation in the use of fluorescent probes to measure reactive oxygen species together with ultrasound. Depending on the ultrasound parameters (frequency and intensity), it may cause chemical reactions in the molecules present in the media, called sonochemistry. When the media are exposed to the ultrasound waves, microbubbles are formed, catastrophically implode and then may interact with the molecules surrounding them, such as the curcumin or the probe, leading to the production of ROS. For this reason, the results obtained in the groups where the ultrasound was applied must be carefully analyzed, since the use of indirect techniques to measure reactive oxygen species have their limitations. In the work performed by Pourhajibagher et al. [42], a nanomicelle curcumin was used for sonodynamic therapy against *Streptococcus mutans*, and the ROS production was measured by using the fluorescent probe 2′,7′-dichlorofluorescein diacetate (H2DCFDA). The authors observed a considerably enhanced ROS generation in the SDI group compared to the control group [42]; however, this probe is not specific to any kind of ROS. For this reason, the authors concluded that one of the mechanisms involved in the bacteria inactivation was ROS production.

**Figure 6:**
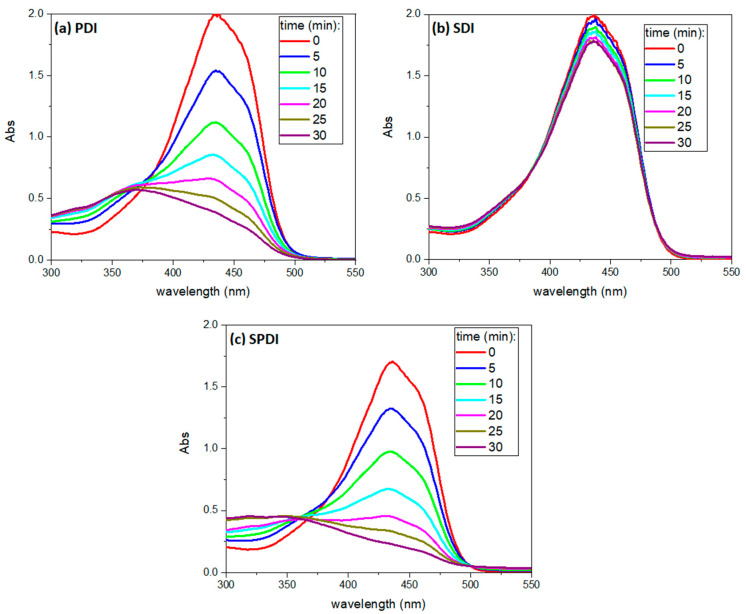
UV-vis representative absorbance spectrum of the curcumin over different sources used in the treatments (SDI—(**a**), PDI—(**b**), SPDI—(**c**)), measured every 5 min until 30 min.

Finally, the absorbance spectrum of Cur was collected every 5 min during the treatments to verify the PS degradation over each source (light, US and light + US) until 30 min, and a representative absorbance spectrum is shown in Figure 6. The curcumin working solution exhibited an absorption maximum of around 435 nm and a small shoulder at 460 nm. After light irradiation (PDI and SPDI), the curcumin absorbance decayed significantly at 435 nm (decay rate of 17 ± 1 and 20 ± 3 min for PDI and SPDI, respectively). However, a slight decay was observed in the SDI group, demonstrating that the degradation of the curcumin by the US at the parameters used was not as expressive as that caused by the light (decay rate of 24 ± 3 min). Additionally, during ultrasound irradiation (SDI and SPDI groups), the absorbance at 250 nm increased with exposure time, and this process did not occur during PDI. For this reason, it was considered a sono-product formed during sonication. The ability of the ultrasound to degrade a sensitizer may be related to the sensitizer characteristic (concentration, solvent and molecule); however, the US parameters (intensity, duty cycle and frequency) also influence it. Ponce et al. [43] performed a comprehensive study of the sono-photo degradation of Protoporphyrin IX (PpIX), where a range of light/US intensities and PpIX concentrations were tested and the PS degradation was monitored. The authors verified that PpIX molecules were degraded by US, light and US + light, and this process was intensified by increasing the intensity of the excitation sources. Also, the absorption spectra revealed that the PpIX decay rate induced by US + light (combined irradiation) was approximately the sum of those induced by photodynamic and sonodynamic activity. Additionally, the authors also observed a sono-product at the region of 250 nm in the same way as observed in the present work [43]. Future works should consider comprehensively evaluating a range of curcumin concentrations, light doses and US intensities to better understand the sonophotochemistry behind SPDI.

The results of the present work contribute to understanding the mechanisms and induced cellular effects of SPDI, which is a new and promising approach for the management of infectious diseases that still has several aspects to be evaluated before being applied clinically. In the literature, the antimicrobial effect of SPDI has been proven against bacteria and fungi using a range of sensitizers. The study by Pourhajibagher et al. [44] assessed the effectiveness of a combined approach involving simultaneous photodynamic and ultrasound intervention (SPDI) in mice infected with *Acinetobacter baumannii*. The therapy was applied with Curcumin-Nisin-based poly (L-lactic acid) nanoparticles (referred to as CurNisNp). The authors observed that as the concentrations of CurNisNp, the duration of light irradiation and the intensity of ultrasound were increased, there was a corresponding dose-dependent decrease in the viability of *A. baumannii* cells. Furthermore, they noted that over time, there was a reduction in the growth of biofilms, alterations in gene expression and an enhancement in wound healing. This was evidenced by the accelerated process of skin re-epithelialization in mice [44]. In another work, Pourhajibagher et al. [45] evaluated SPDI using hypericin nanoparticles (HypNP) in combination with D-Tryptophan (D-Trp) against *A. baumannii*. The cell viability of bacterial cells experienced a remarkable decrease of 5.10 logarithmic units when subjected to a treatment involving half of the minimum inhibitory concentration (1/2 × MIC) of HypNP@D-Trp, followed by exposure to ultrasound waves and blue light. Furthermore, there was a notable decrease in the viability of *A. baumannii* biofilm upon treatment with SPDI using double the minimum inhibitory concentration (2 × MIC) of HypNP@D-Trp, especially when compared to the control groups [45]. In the study of Xu et al. [46], the authors evaluated the synergistic effects of ultrasonic irradiation combined with photodynamic inactivation (PDI) to improve the efficacy of PDI against a methicillin-resistant *Staphylococcus aureus* (MRSA) biofilm. They applied a cationic benzylidene cyclopentanone as a photosensitizer in their study. Their findings revealed that the percentage decrease in metabolic activity observed in the group subjected to both ultrasonic irradiation and PDI (US + PDI group) was notably higher, reaching 75.76%, in comparison to the combined effects of the PDI group (44.14%) and the US group (9.88%). This outcome suggests a synergistic interaction between ultrasound and photodynamic intervention [46]. It is important to emphasize that those studies were performed with SPDI mediated by newly synthesized sensitizers, and in the present work, curcumin, a conventional sensitizer, was employed and exhibited promising results, demonstrating that SPDI may be applied using a traditional and natural sensitizer. Interestingly, Ziental et al. [47] evaluated two popular and well-researched dyes, chlorin e6 (Ce6) and rose bengal (RB), and the sensitizers were excited by using two different methods (ultrasound and light) against methicillin-resistant *Staphylococcus aureus.* In this study, the authors showed that RB was a more efficient sonosensitizer than Ce6. The dual synergism between RB and Ce6 was noticed, achieving a >3 log reduction for molar ratios RB:Ce6 of 1:1 and 1:3, while, alone, the sensitizers excited with ultrasound and light achieved only ca. a 1 log reduction [47]. Therefore, these findings together with the results obtained in the present work suggest that the effects of the combined treatment are greater for biofilm inactivation.

## 4. Conclusions

Taking all of the results into account, it was possible to conclude that the SPDI was more effective in inactivating the bacteria biofilm than PDI and SDI when mediated by curcumin. Treatments led to a decrease in the bacteria’s capacity to adhere, probably linked to the reduction in cellular metabolism within the treated groups. In terms of the ability to form biofilms, the treatments did not result in a CFU/mL reduction; nevertheless, the biofilm showed lower biomass, indicating that PDI, SDI and SPDI altered the characteristics of the biofilm morphology. It is possible to suggest that this thinner biofilm would be more susceptible to a second session of treatment. Besides that, one possible mechanism involved in the inactivation was the ROS production observed, where singlet oxygen was detected in the groups to which the light was applied and hydroxyl radicals were collected in the groups where the US was used. The curcumin had a significant decay in its absorbance with the application of light, and the degradation caused by the US was lesser compared to the illuminated groups. Finally, SPDI significantly reduced the cells and ECM of the biofilm and impacted its structure. In conclusion, SPDI was more effective in *S. aureus* inactivation; however, all treatments had the same impact on the virulence factors evaluated, making the biofilm more susceptible to a second session of treatment. Future works should consider performing a study evaluating a range of curcumin concentrations, light doses and US intensities to verify the PS behavior over different conditions of treatment.

## Figures and Tables

**Table 1:** Mean values (in percentage) and standard deviation of the adhesion capacity, biofilm formation ability reduction (total biomass) and metabolic activity reduction of the Control, PDI, SDI and SPDI groups.

	Control	PDI	SDI	SPDI
Adhesion capacity	79 ± 1%	71 ± 1%	58 ± 1%	58 ± 2%
Biofilm formation ability reduction (total biomass)	10 ± 1%	26 ± 2%	31 ± 5%	35 ± 6%
Metabolic activity reduction	-	89 ± 1%	82 ± 2%	90 ± 1%

**Table 2:** Mean values and standard deviation of the fluorescence intensities of the test groups immediately after the treatments, equivalent to the production of the hydroxyl radicals (APF probe, emission at 515 nm) and singlet oxygen species (SOSG probe, emission at 525 nm).

	Probe	Cur	Light	US	PDI	SDI	SPDI
Hydroxyl Radicals (APF)	20,900 ± 400	3700 ± 900	8300 ± 500	150,000 ± 10,000	36,000 ± 1000	72,000 ± 2000	42,000 ± 2000
Singlet Oxygen Species (SOSG)	5000 ± 20,000	30,000 ± 10,000	650,000 ± 20,000	100,000 ± 40,000	460,000 ± 10,000	40,000 ± 20,000	430,000 ± 20,000

## Data Availability

Not applicable.

## Supplementary Materials

The following supporting information can be downloaded at: https://www.mdpi.com/article/10.3390/pharmaceutics15102407/s1, Figure S1: Raw Confocal Images obtained of the biofilms from the control, light, US, PDI, SDI and SPDI groups. Biofilms were stained with LIVE/DEAD (SYTO 9 and Propidium Iodide), which mark viable (green color) and nonviable cells (red color). The ECM was stained with Calcofluor White (blue color). The stained biofilms were imaged, using as excitation/emission wavelengths at 480/500 nm for SYTO-9 stain, 490/635 nm for PI and 405/433 nm for Calcofluor, as recommended by the manufacturers. The green boxes are transversal images showing the thickness of the biofilms., Figure S2: Raw OCT images obtained from the Control, light US, US+Light, PDI, SDI and SPDI groups. Images were obtained with medium sensitivity, speed of 76 kHz, with field image correction, subsampling filter, scanning pattern of 400 × 400 × 512 pixels (X, Y, Z) and pixel size of 5 × 5 × 2.49 μm (X, Y, Z). Transversal images of the biofilms were obtained to evaluate the cell density, thickness and topography of the biofilm.

## References

[1] Lowy F.D. (1998). Staphylococcus aureus infections. N. Engl. J. Med..

[2] Sharma A.K., Dhasmana N., Dubey N., Kumar N., Gangwal A., Gupta M., Singh Y. (2017). Bacterial Virulence Factors: Secreted for Survival. Indian J. Microbiol..

[3] Muhammad M.H., Idris A.L., Fan X., Guo Y., Yu Y., Jin X., Qiu J., Guan X., Huang T. (2020). Beyond Risk: Bacterial Biofilms and Their Regulating Approaches. Front. Microbiol..

[4] Yin W., Wang Y., Liu L., He J. (2019). Biofilms: The Microbial “Protective Clothing” in Extreme Environments. Int. J. Mol. Sci..

[5] Jamal M., Ahmad W., Andleeb S., Jalil F., Imran M., Nawaz M.A., Hussain T., Ali M., Rafiq M., Kamil M.A. (2018). Bacterial biofilm and associated infections. J. Chin. Med. Assoc..

[6] Can Karanlık C., Karanlık G., Özdemir S., Tollu G., Erdoğmuş A. (2023). Synthesis and characterization of novel BODIPYs and their antioxidant, antimicrobial, photodynamic antimicrobial, antibiofilm and DNA interaction activities. Photochem. Photobiol..

[7] Sellera F.P., Sabino C.P., Hamblin M.R., Sellera F.P., Nascimento C.L., Ribeiro M.S. (2016). Photodynamic Therapy in Veterinary Medicine: From Basics to Clinical Practice.

[8] Vera D.M., Haynes M.H., Ball A.R., Dai T., Astrakas C., Kelso M.J., Hamblin M.R., Tegos G.P. (2012). Strategies to potentiate antimicrobial photoinactivation by overcoming resistant phenotypes. Photochem. Photobiol..

[9] Wainwright M., Maisch T., Nonell S., Plaetzer K., Almeida A., Tegos G.P., Hamblin M.R. (2017). Photoantimicrobials-are we afraid of the light?. Lancet Infect. Dis..

[10] Maisch T., Baier J., Franz B., Maier M., Landthaler M., Szeimies R.M., Bäumler W. (2007). The role of singlet oxygen and oxygen concentration in photodynamic inactivation of bacteria. Proc. Natl. Acad. Sci. USA.

[11] Alves E., Faustino M.A., Neves M.G., Cunha A., Tome J., Almeida A. (2014). An insight on bacterial cellular targets of photodynamic inactivation. Future Med. Chem..

[12] Nakonechny F., Nisnevitch M., Nitzan Y., Nisnevitch M. (2013). Sonodynamic excitation of Rose Bengal for eradication of gram-positive and gram-negative bacteria. Biomed Res. Int..

[13] Alves F., Pavarina A.C., Mima E.G.O., McHale A.P., Callan J.F. (2018). Antimicrobial sonodynamic and photodynamic therapies against Candida albicans. Biofouling.

[14] Alaqeel S.M., Moussa I.M., Altinawi A., Almozainy M., Hashem M. (2023). Antibacterial Effectiveness of Photo-Sonodynamic Treatment by Methylene Blue-incorporated Poly(D, L-Lactide-Co-Glycolide) Acid Nanoparticles to Disinfect Root Canals. Photodiagn. Photodyn. Ther..

[15] Alves F., Gomes Guimarães G., Mayumi Inada N., Pratavieira S., Salvador Bagnato V., Kurachi C. (2021). Strategies to Improve the Antimicrobial Efficacy of Photodynamic, Sonodynamic, and Sonophotodynamic Therapies. Lasers Surg. Med..

[16] Wysocki M., Czarczynska-Goslinska B., Ziental D., Michalak M., Güzel E., Sobotta L. (2022). Excited State and Reactive Oxygen Species against Cancer and Pathogens: A Review on Sonodynamic and Sono-Photodynamic Therapy. ChemMedChem.

[17] Hiraoka W., Honda H., Feril L.B., Kudo N., Kondo T. (2006). Comparison between sonodynamic effect and photodynamic effect with photosensitizers on free radical formation and cell killing. Ultrason. Sonochem..

[18] Alves F., Ayala E.T.P., Pratavieira S. (2021). Sonophotodynamic Inactivation: The power of light and ultrasound in the battle against microorganisms. J. Photochem. Photobiol..

[19] Kenyon J.N., Fuller R.J., Lewis T.J. (2009). Activated cancer therapy using light and ultrasound—A case series of sonodynamic photodynamic therapy in 115 patients over a 4 year period. Curr. Drug Ther..

[20] Zheng Y., Ye J., Li Z., Chen H., Gao Y. (2021). Recent progress in sono-photodynamic cancer therapy: From developed new sensitizers to nanotechnology-based efficacy-enhancing strategies. Acta Pharm. Sin. B.

[21] Sadanala K.C., Chaturvedi P.K., Seo Y.M., Kim J.M., Jo Y.S., Lee Y.K., Ahn W.S. (2014). Sono-photodynamic combination therapy: A review on sensitizers. Anticancer Res..

[22] Yang Y., Tu J., Yang D., Raymond J.L., Roy R.A., Zhang D. (2019). Photo- and Sono-Dynamic Therapy: A Review of Mechanisms and Considerations for Pharmacological Agents Used in Therapy Incorporating Light and Sound. Curr. Pharm. Des..

[23] Nene L.C., Nyokong T. (2023). The in-vitro proliferation-suppression of MCF-7 and HeLa cell lines mediated by differently substituted ionic phthalocyanines in sonodynamic therapy supplemented-photodynamic therapy. J. Inorg. Biochem..

[24] Niavarzi S., Pourhajibagher M., Khedmat S., Ghabraei S., Chiniforush N., Bahador A. (2019). Effect of ultrasonic activation on the efficacy of antimicrobial photodynamic therapy: Evaluation of penetration depth of photosensitizer and elimination of Enterococcus faecalis biofilms. Photodiagn. Photodyn. Ther..

[25] Pourhajibagher M., Bahador A. (2021). Attenuation of Aggregatibacter actinomycetemcomitans virulence using curcumin-decorated nanophytosomes-mediated photo-sonoantimicrobial chemotherapy. Sci. Rep..

[26] Pourhajibagher M., Rokn A.R., Barikani H.R., Bahador A. (2020). Photo-sonodynamic antimicrobial chemotherapy via chitosan nanoparticles-indocyanine green against polymicrobial periopathogenic biofilms: Ex vivo study on dental implants. Photodiagn. Photodyn. Ther..

[27] Liu T.Y., Tan Z.J., Jiang L., Gu J.F., Wu X.S., Cao Y., Li M.L., Wu K.J., Liu Y.B. (2013). Curcumin induces apoptosis in gallbladder carcinoma cell line GBC-SD cells. Cancer Cell Int..

[28] Obeid M.A., Alsaadi M., Aljabali A.A. (2023). Recent updates in curcumin delivery. J. Liposome Res..

[29] Li X., Liu Z., Liu H., Chen X., Liu Y., Tan H. (2017). Photodynamic inactivation of fibroblasts and inhibition of Staphylococcus epidermidis adhesion and biofilm formation by toluidine blue O. Mol. Med. Rep..

[30] Alves F., de Oliveira Mima E.G., Passador R.C.P., Bagnato V.S., Jorge J.H., Pavarina A.C. (2017). Virulence factors of fluconazole-susceptible and fluconazole-resistant Candida albicans after antimicrobial photodynamic therapy. Lasers Med. Sci..

[31] Soares B.M., da Silva D.L., Sousa G.R., Amorim J.C., de Resende M.A., Pinotti M., Cisalpino P.S. (2009). In vitro photodynamic inactivation of Candida spp. growth and adhesion to buccal epithelial cells. J. Photochem. Photobiol. B.

[32] Carmello J.C., Alves F., Basso F.G., de Souza Costa C.A., Tedesco A.C., Lucas Primo F., Mima E.G.O., Pavarina A.C. (2019). Antimicrobial photodynamic therapy reduces adhesion capacity and biofilm formation of Candida albicans from induced oral candidiasis in mice. Photodiagn. Photodyn. Ther..

[33] da Silva B.G.M., Carvalho M.L., Rosseti I.B., Zamuner S., Costa M.S. (2018). Photodynamic antimicrobial chemotherapy (PACT) using toluidine blue inhibits both growth and biofilm formation by Candida krusei. Lasers Med. Sci..

[34] Fekrirad Z., Kashef N., Arefian E. (2019). Photodynamic inactivation diminishes quorum sensing-mediated virulence factor production and biofilm formation of Serratia marcescens. World J. Microbiol. Biotechnol..

[35] Pourhajibagher M., Chiniforush N., Shahabi S., Ghorbanzadeh R., Bahador A. (2016). Sub-lethal doses of photodynamic therapy affect biofilm formation ability and metabolic activity of Enterococcus faecalis. Photodiagn. Photodyn. Ther..

[36] Obeng E., Feng J., Wang D., Zheng D., Xiang B., Shen J. (2022). Multifunctional phototheranostic agent ZnO@Ag. for anti-infection through photothermal/photodynamic therapy. Front. Chem..

[37] Karanlık C.C., Aguilar-Galindo F., Sobotta L., Güzel E., Erdoğmuş A. (2023). Combination of Light and Ultrasound: Exploring Sono–Photochemical Activities of Phthalocyanine-Based Sensitizers. J. Phys. Chem. C.

[38] Wang R., Liu Q., Gao A., Tang N., Zhang Q., Zhang A., Cui D. (2022). Recent developments of sonodynamic therapy in antibacterial application. Nanoscale.

[39] Choi V., Rajora M.A., Zheng G. (2020). Activating Drugs with Sound: Mechanisms behind Sonodynamic Therapy and the Role of Nanomedicine. Bioconjug Chem..

[40] Perry E.K., Tan M.W. (2023). Bacterial biofilms in the human body: Prevalence and impacts on health and disease. Front. Cell Infect. Microbiol..

[41] Ali A., Zahra A., Kamthan M., Husain F.M., Albalawi T., Zubair M., Alatawy R., Abid M., Noorani M.S. (2023). Microbial Biofilms: Applications, Clinical Consequences, and Alternative Therapies. Microorganisms.

[42] Pourhajibagher M., Rahimi Esboei B., Hodjat M., Bahador A. (2020). Sonodynamic excitation of nanomicelle curcumin for eradication of Streptococcus mutans under sonodynamic antimicrobial chemotherapy: Enhanced anti-caries activity of nanomicelle curcumin. Photodiagn. Photodyn. Ther..

[43] Ponce Ayala E.T., Alves Dias de Sousa F., Vollet-Filho J.D., Rodrigues Garcia M., de Boni L., Salvador Bagnato V., Pratavieira S. (2021). Photodynamic and Sonodynamic Therapy with Protoporphyrin IX: In Vitro and In Vivo Studies. Ultrasound Med. Biol..

[44] Pourhajibagher M., Pourakbari B., Bahador A. (2022). Contribution of antimicrobial photo-sonodynamic therapy in wound healing: An in vivo effect of curcumin-nisin-based poly (L-lactic acid) nanoparticle on Acinetobacter baumannii biofilms. BMC Microbiol..

[45] Pourhajibagher M., Hosseini N., Bahador A. (2023). Antimicrobial activity of D-amino acid in combination with photo-sonoactivated hypericin nanoparticles against Acinetobacter baumannii. BMC Microbiol..

[46] Xu Y., Liu S., Zhao H., Li Y., Cui C., Chou W., Zhao Y., Yang J., Qiu H., Zeng J. (2023). Ultrasonic irradiation enhanced the efficacy of antimicrobial photodynamic therapy against methicillin-resistant Staphylococcus aureus biofilm. Ultrason. Sonochem..

[47] Ziental D., Wysocki M., Michalak M., Dlugaszewska J., Güzel E., Sobotta L. (2023). The Dual Synergy of Photodynamic and Sonodynamic Therapy in the Eradication of Methicillin-Resistant *Staphylococcus aureus*. Appl. Sci..

