# Supplementary material for: Effects on Colonization Factors and Mechanisms Involved in Antimicrobial Sonophotodynamic Inactivation Mediated by Curcumin
Source: Pharmaceutics. 2023 Sep 30;15(10):2407. doi: 10.3390/pharmaceutics15102407 (PMC10610509; doi:10.3390/pharmaceutics15102407)

Figure S1. Raw Confocal Images obtained of the biofilms from the control, light, US, PDI, SDI and SPDI groups. Biofilms were stained with LIVE/DEAD (SYTO 9 and Propidium Iodide), which mark viable (green color) and nonviable cells (red color). The ECM was stained with Calcofluor White (blue color). The stained biofilms were imaged, using as excitation/emission wavelengths at 480/500 nm for SYTO-9 stain, 490/635 nm for PI and 405/433 nm for Calcofluor, as recommended by the manufacturers. The green boxes are transversal images showing the thickness of the biofilms.

Control

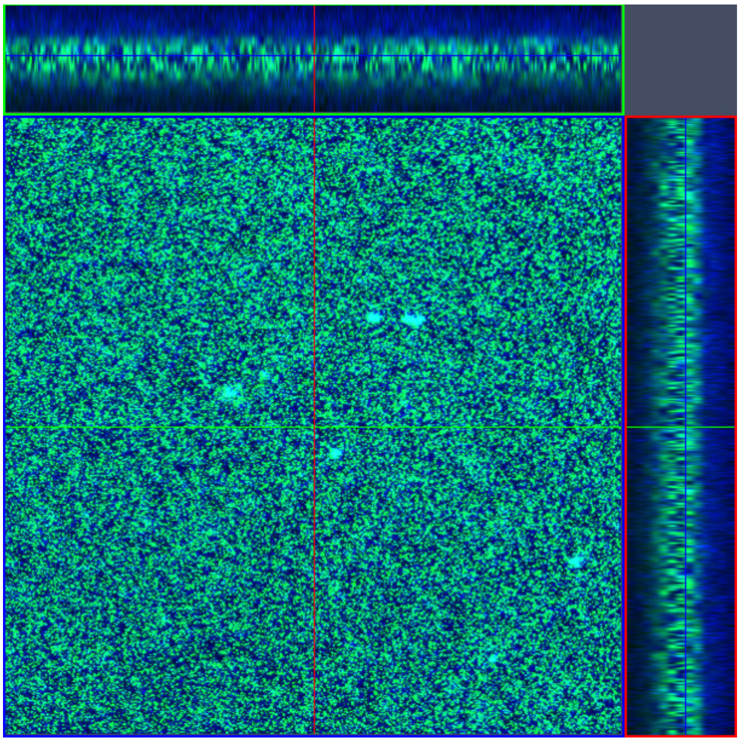

Light

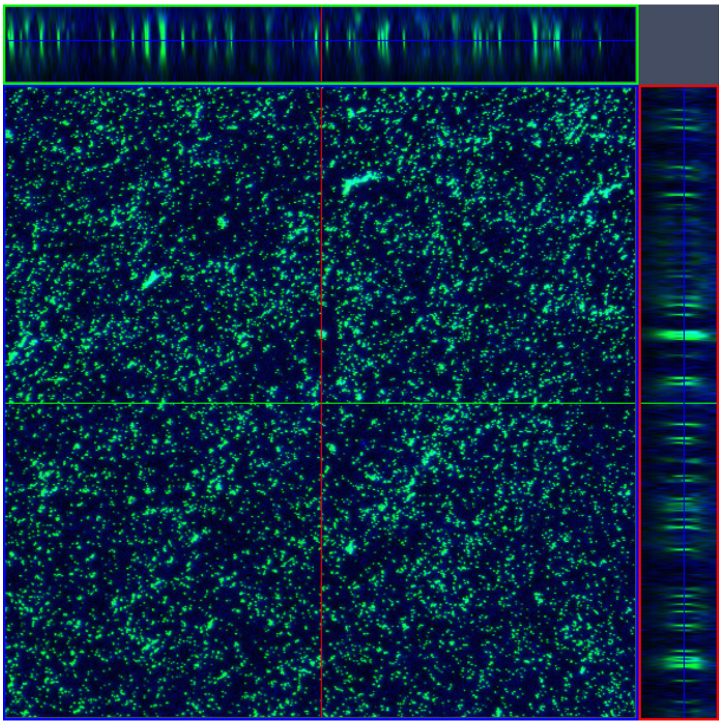

US

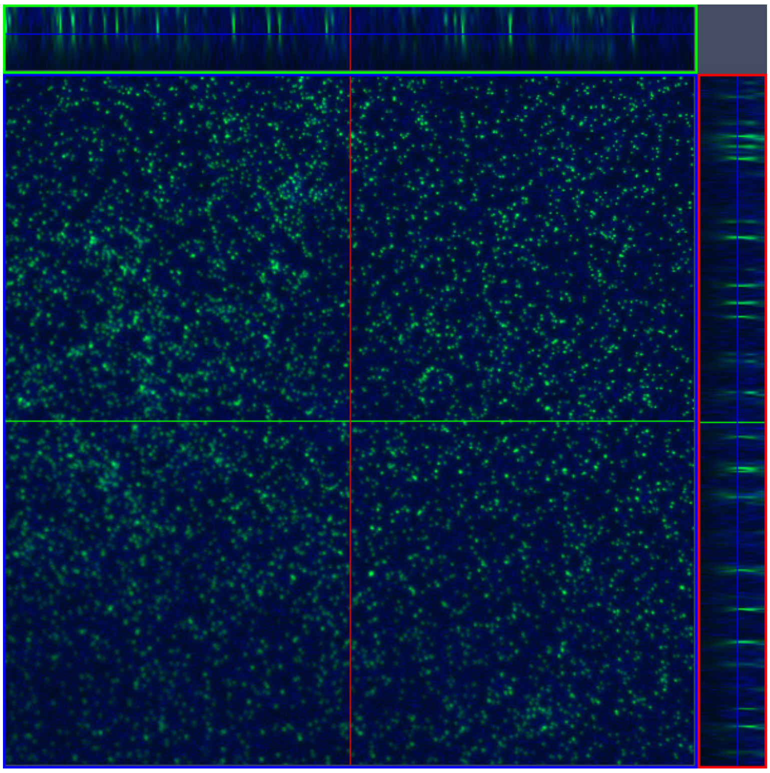

PDI

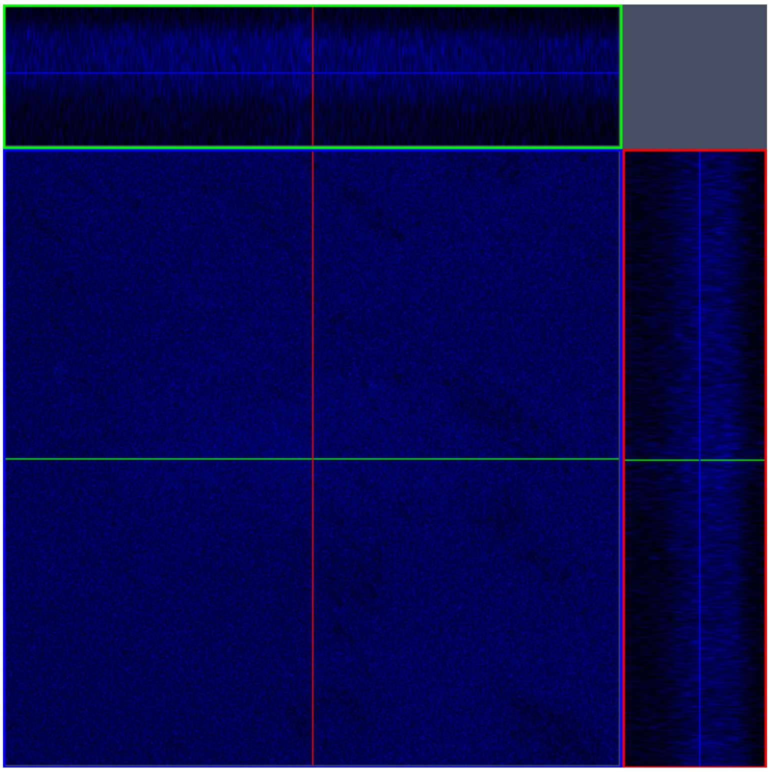

SDI

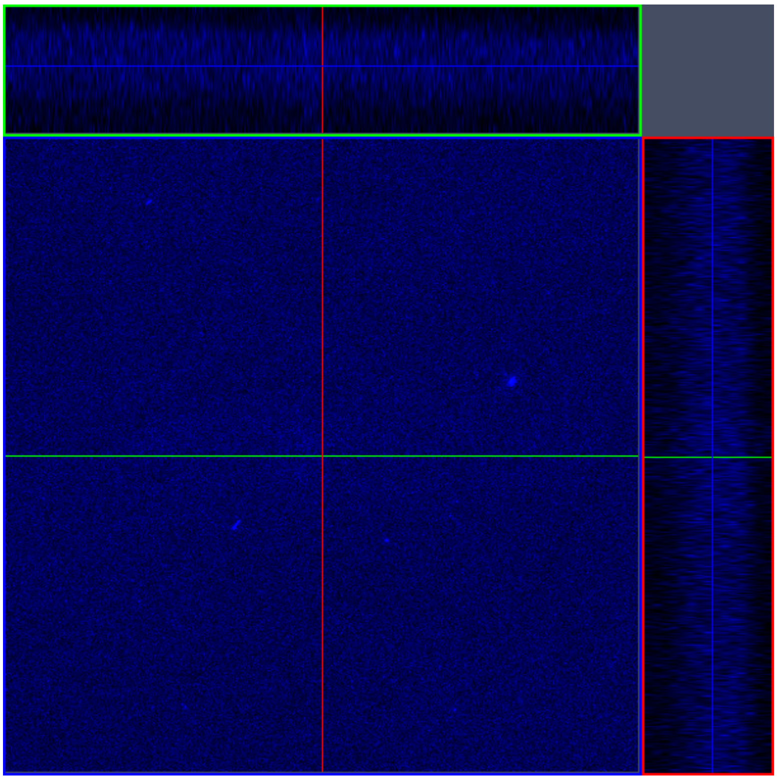

SPDI

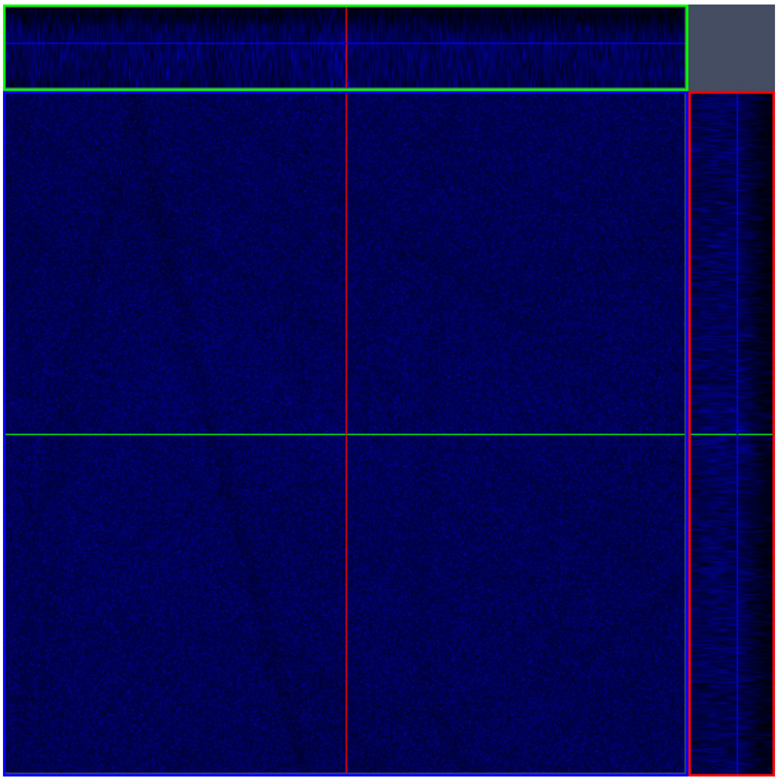

Figure S2. Raw OCT images obtained from the Control, light US, US+Light, PDI, SDI and SPDI groups. Images were obtained with medium sensitivity, speed of 76 kHz, with field image correction, subsampling filter, scanning pattern of 400 x 400 x 512 pixels (X, Y, Z) and pixel size of 5 x 5 x 2.49 μm (X, Y, Z). Transversal images of the biofilms were obtained to evaluate the cell density, thickness and topography of the biofilm.

Control

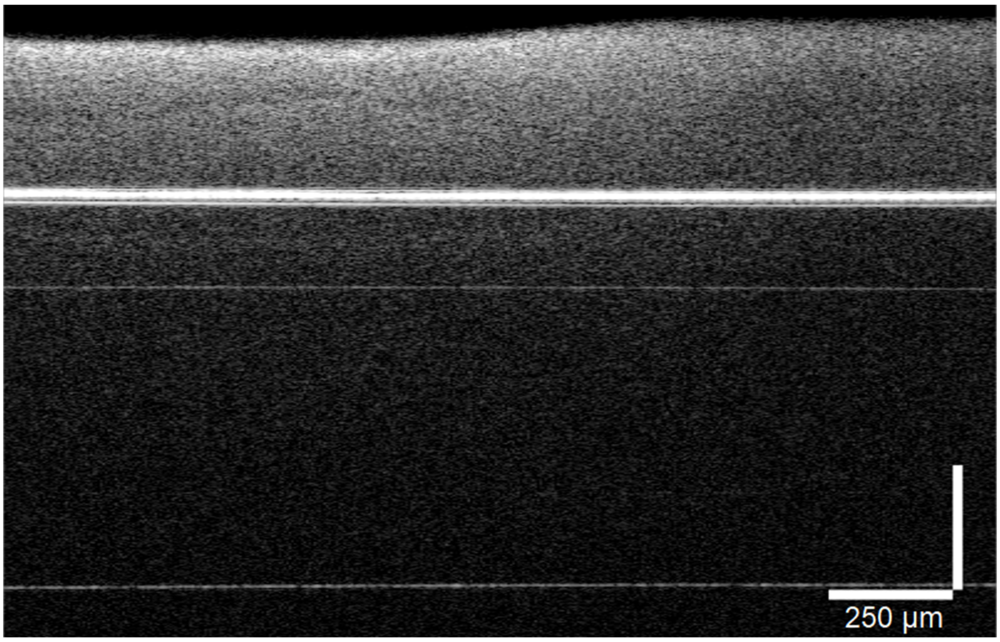

Light

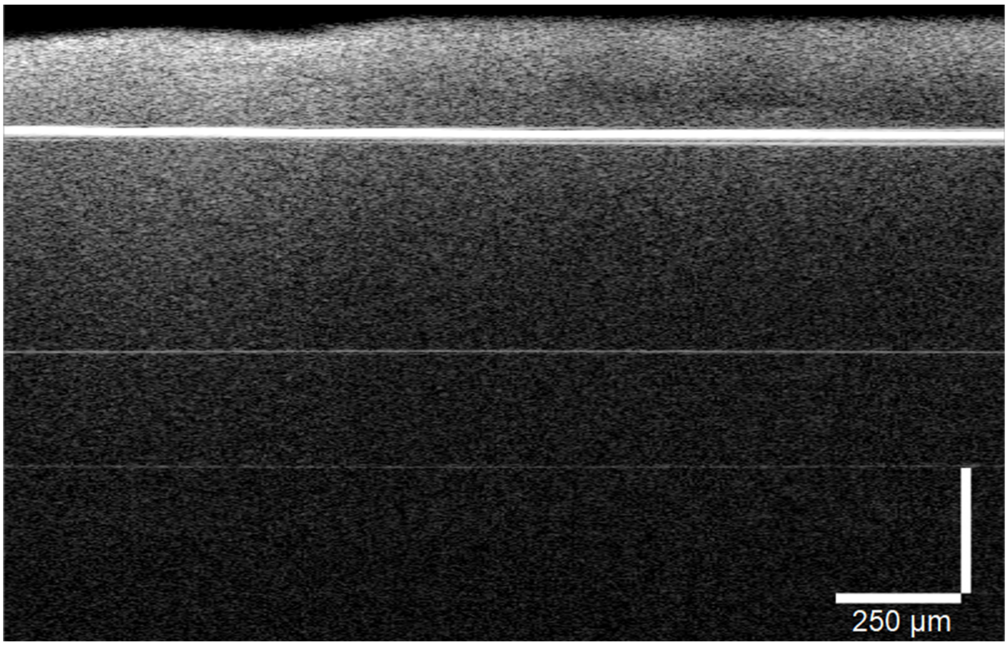

US

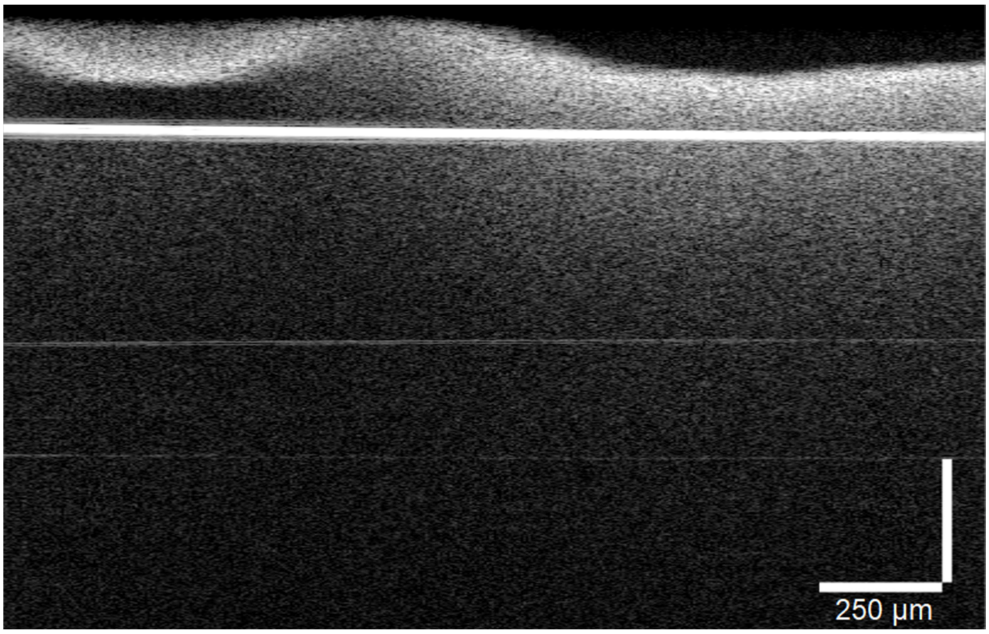

Light+US

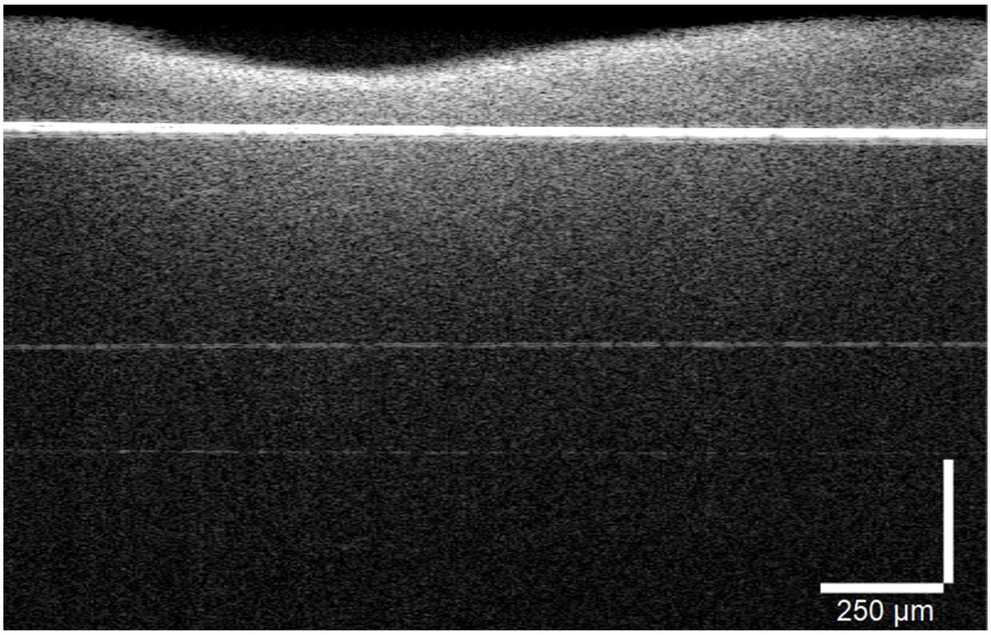

PDI

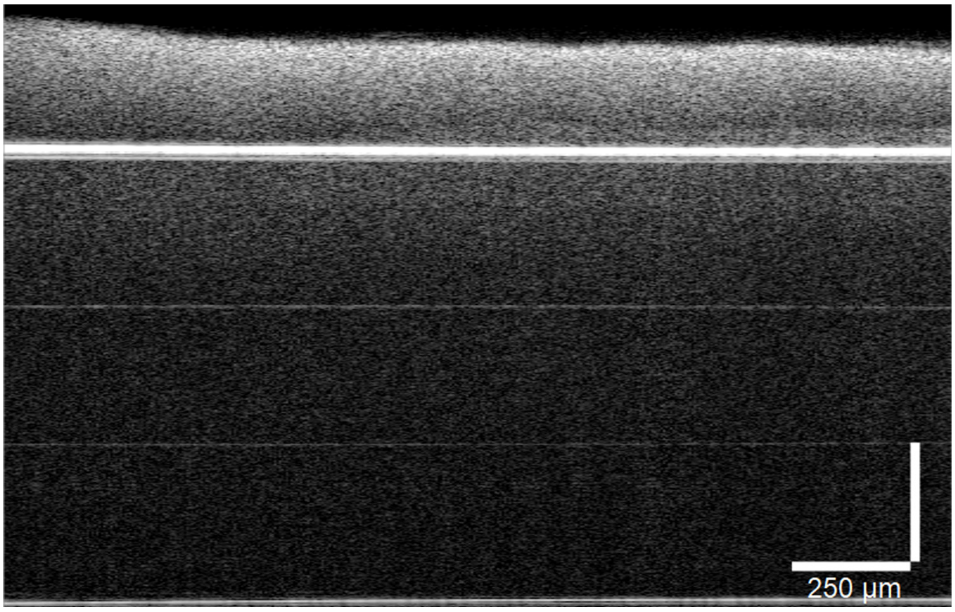

SDI

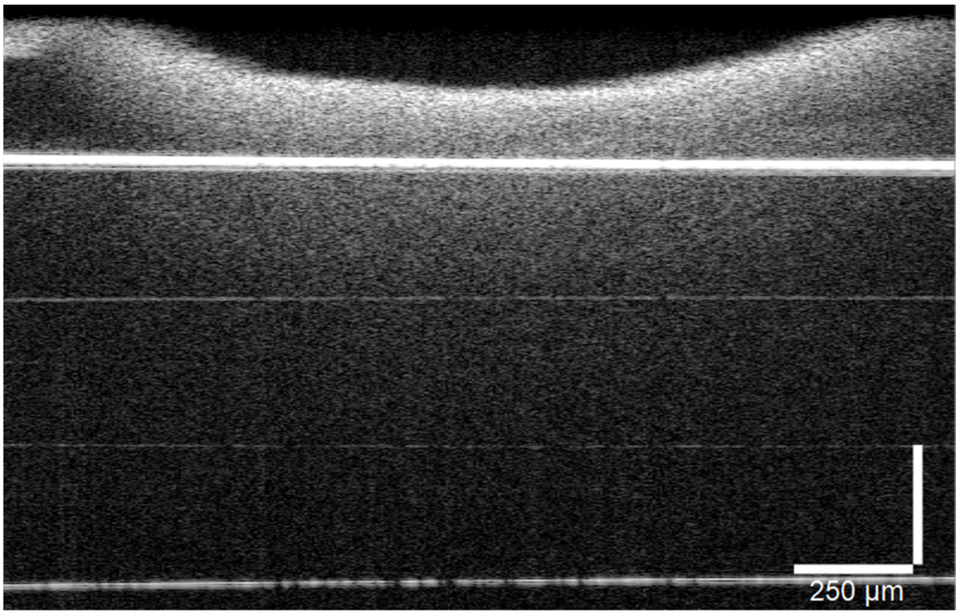

SPDI

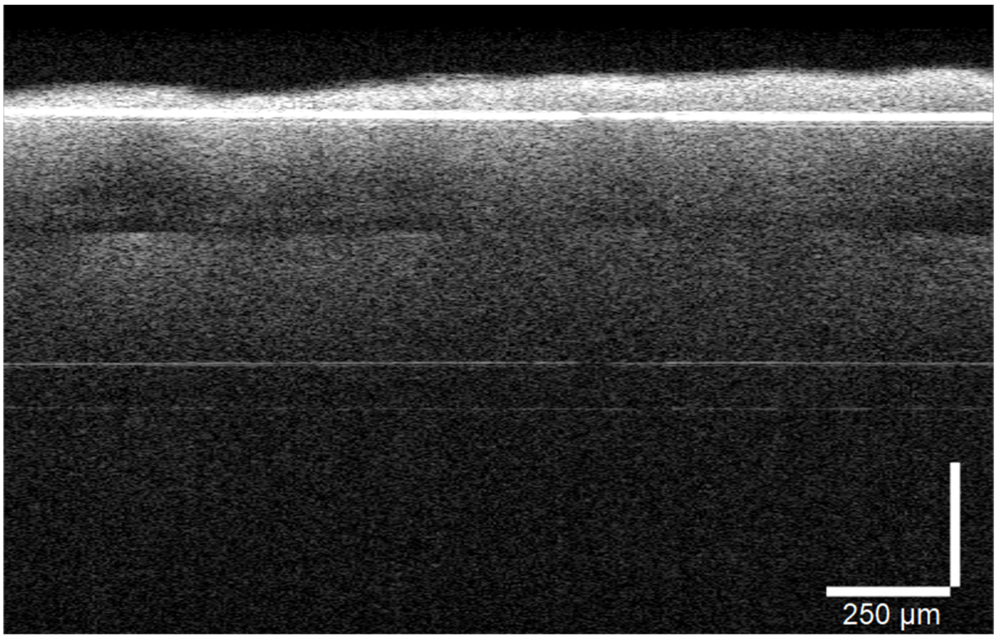

Supplement: Supplementary file 1 [file pharmaceutics-15-02407-s001.zip › Supplementary images.pdf]
