# Supplementary figures and images for: Effects on Colonization Factors and Mechanisms Involved in Antimicrobial Sonophotodynamic Inactivation Mediated by Curcumin
Source: Pharmaceutics. 2023 Sep 30;15(10):2407. doi: 10.3390/pharmaceutics15102407 (PMC10610509; doi:10.3390/pharmaceutics15102407)

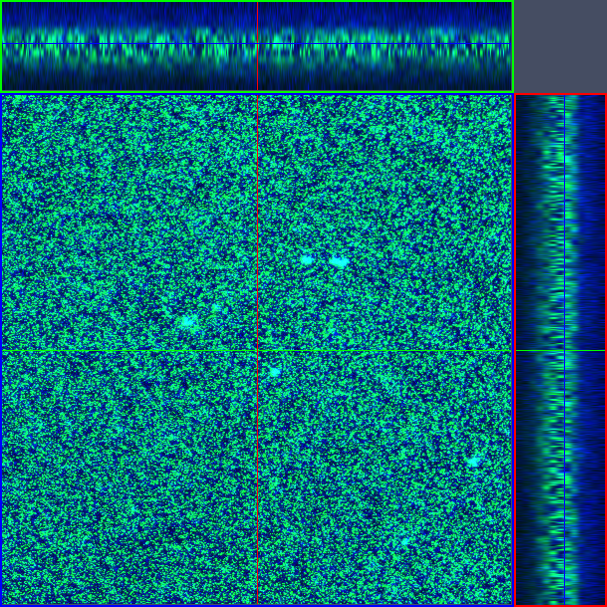

Supplement: Supplementary file 1 [file pharmaceutics-15-02407-s001.zip › Confocal original images/Control.tif]

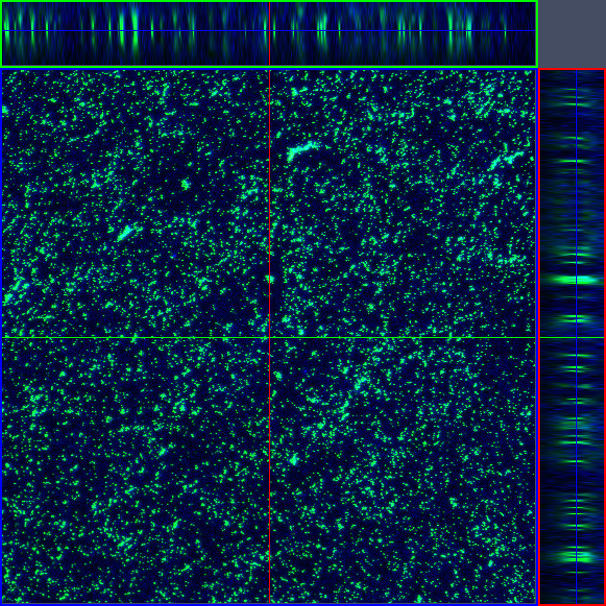

Supplement: Supplementary file 1 [file pharmaceutics-15-02407-s001.zip › Confocal original images/Light.tif]

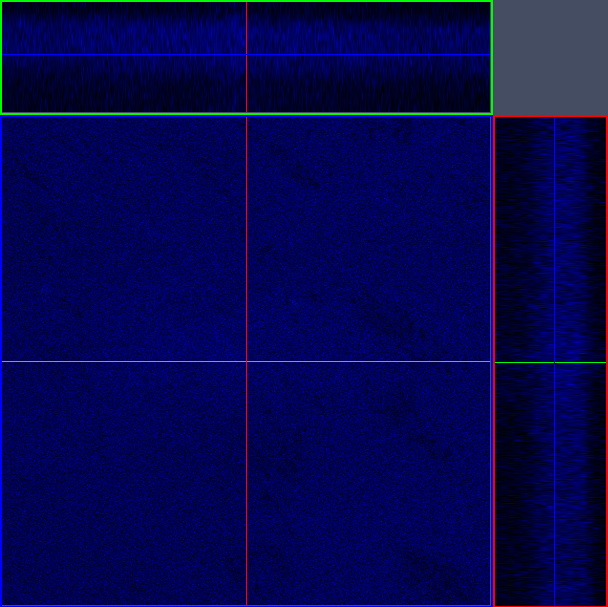

Supplement: Supplementary file 1 [file pharmaceutics-15-02407-s001.zip › Confocal original images/PDI.tif]

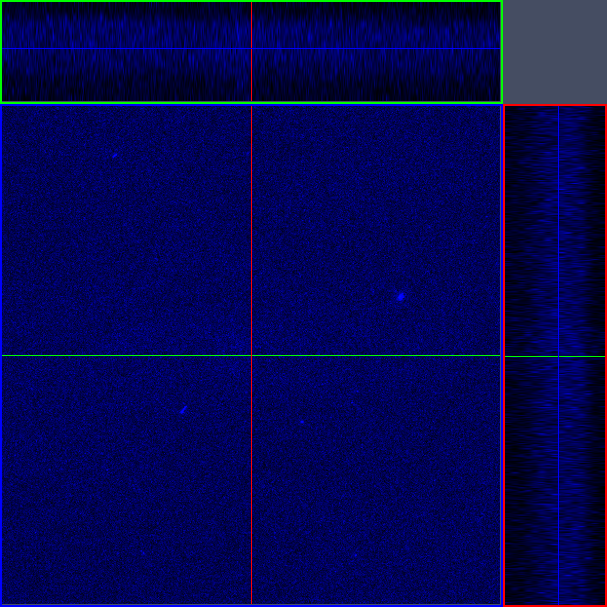

Supplement: Supplementary file 1 [file pharmaceutics-15-02407-s001.zip › Confocal original images/SDI.tif]

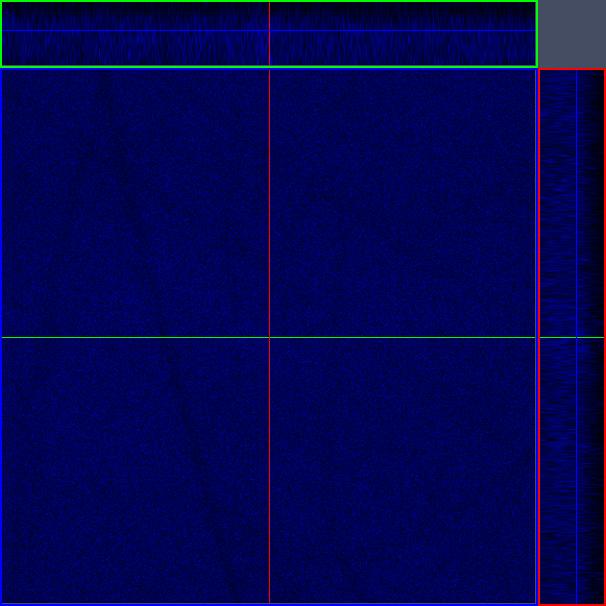

Supplement: Supplementary file 1 [file pharmaceutics-15-02407-s001.zip › Confocal original images/SPDI.tif]

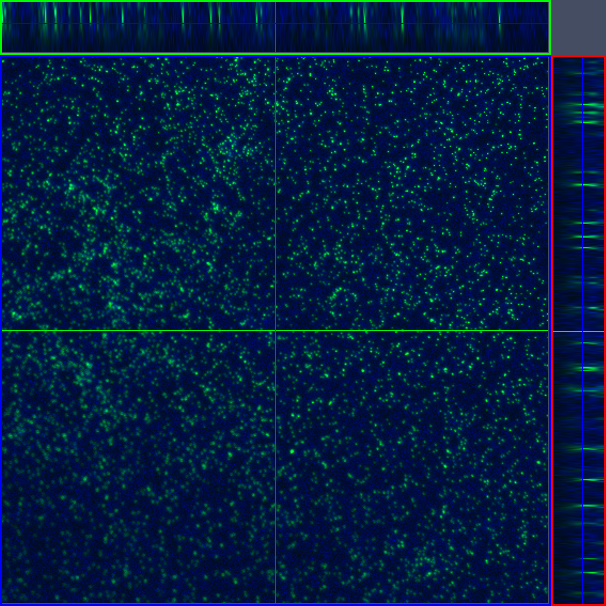

Supplement: Supplementary file 1 [file pharmaceutics-15-02407-s001.zip › Confocal original images/US.tif]

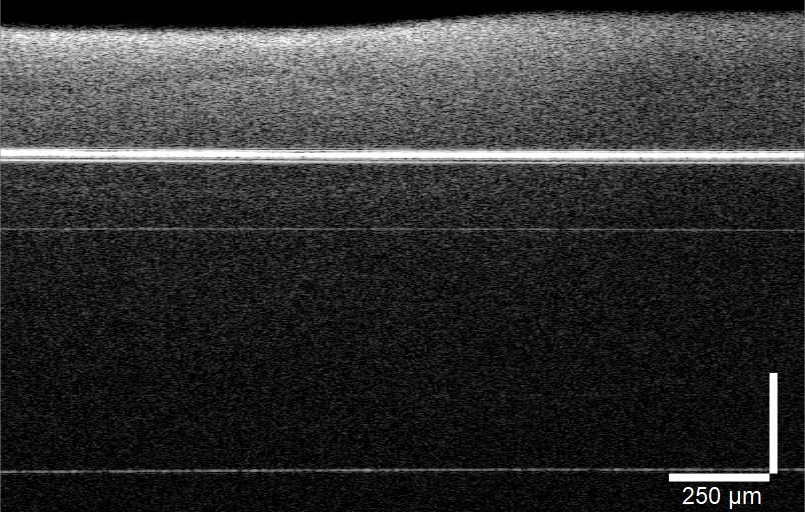

Supplement: Supplementary file 1 [file pharmaceutics-15-02407-s001.zip › OCT original images/Control.tiff]

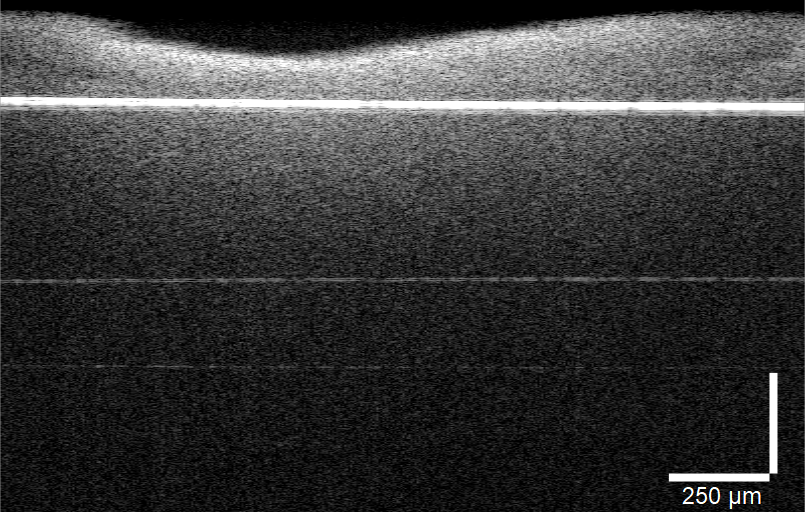

Supplement: Supplementary file 1 [file pharmaceutics-15-02407-s001.zip › OCT original images/Light+US.tiff]

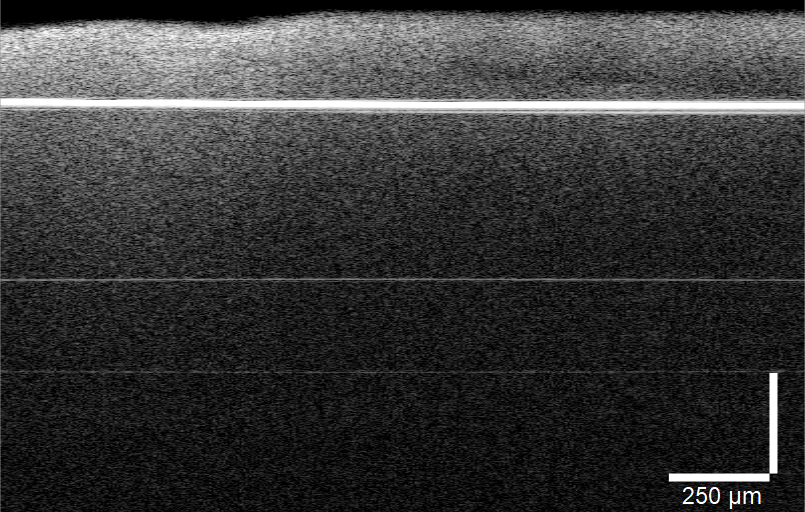

Supplement: Supplementary file 1 [file pharmaceutics-15-02407-s001.zip › OCT original images/Light.tiff]

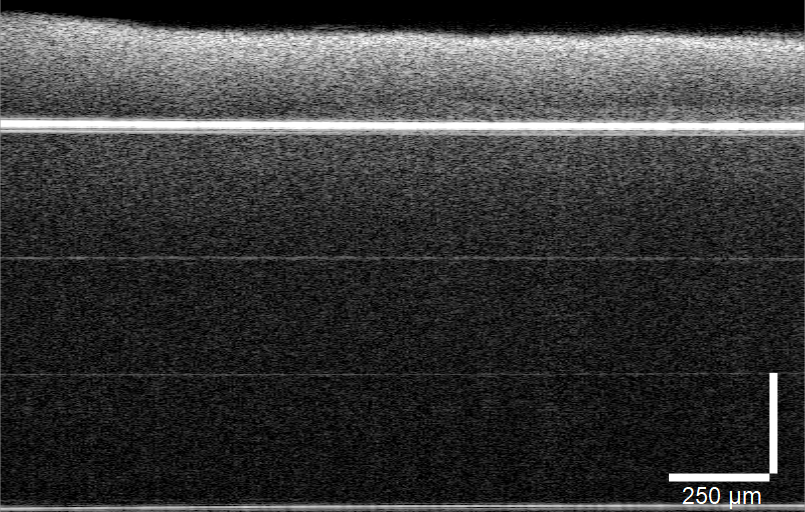

Supplement: Supplementary file 1 [file pharmaceutics-15-02407-s001.zip › OCT original images/PDI.tiff]

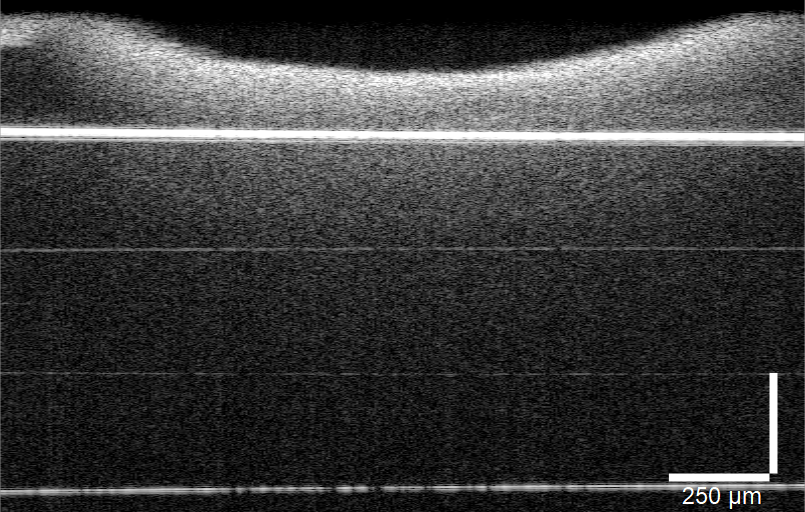

Supplement: Supplementary file 1 [file pharmaceutics-15-02407-s001.zip › OCT original images/SDI.tiff]

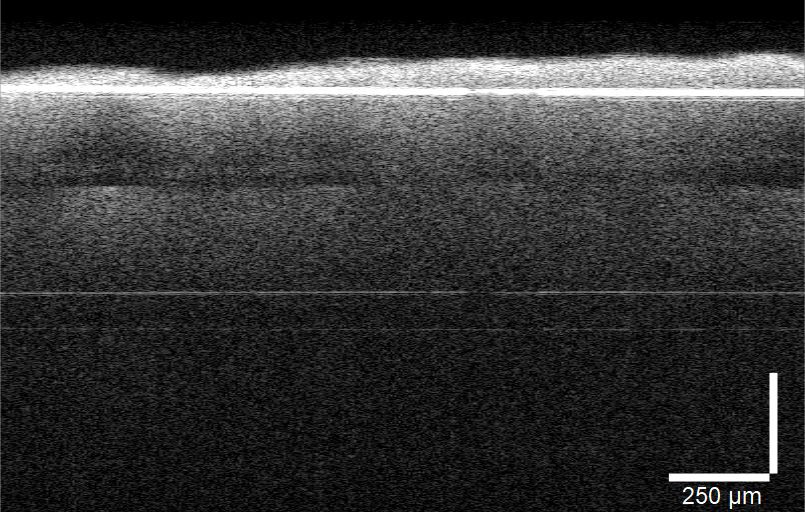

Supplement: Supplementary file 1 [file pharmaceutics-15-02407-s001.zip › OCT original images/SPDI.tiff]

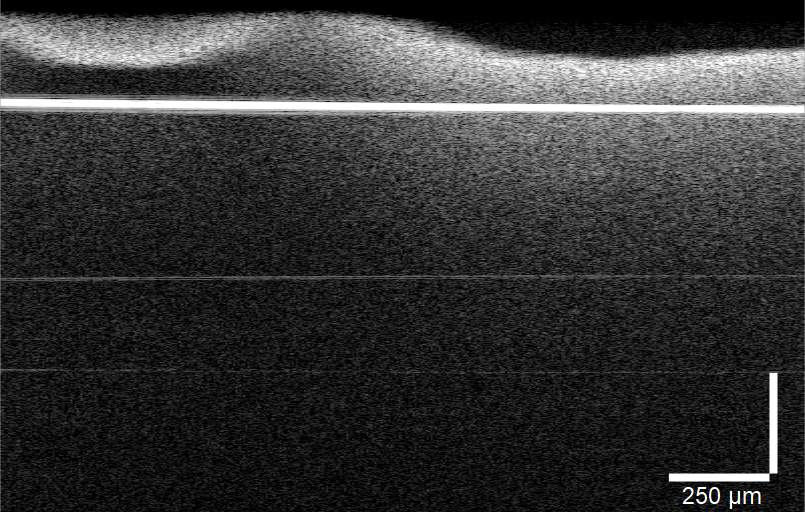

Supplement: Supplementary file 1 [file pharmaceutics-15-02407-s001.zip › OCT original images/US.tiff]
